# Systematic elucidation of the effective constituents and potential mechanisms of *Scrophulariae Radix* against neoplasm based on LC-MS, network pharmacology, and molecular docking approaches

**DOI:** 10.3389/fpls.2025.1615076

**Published:** 2025-07-03

**Authors:** Shu-jie Yu, Xiao-bin Kong, Xin Jin, Meng-yi Shan, Gang Cheng, Pei-lu Wang, Wen-long Li, Pei-yuan Zhao, Yun-jie Sheng, Bing-qian He, Qi Shi, Hua-qiang Li, Qi-ming Zhao, Lu-ping Qin, Xiong-yu Meng

**Affiliations:** ^1^ School of Pharmaceutical Sciences, Zhejiang Chinese Medical University, Hangzhou, Zhejiang, China; ^2^ School of Basic Medical Sciences, Zhejiang Chinese Medical University, Hangzhou, Zhejiang, China; ^3^ Institute of Interdisciplinary Integrative Medicine Research, Shanghai University of Traditional Chinese Medicine, Shanghai, China; ^4^ Academy of Chinese Medical Science, Zhejiang Chinese Medical University, Hangzhou, Zhejiang, China; ^5^ Zhejiang Key Laboratory of Chinese Medicine Modernization, Zhejiang Chinese Medical University, Hangzhou, Zhejiang, China

**Keywords:** *Scrophulariae Radix*, neoplasm, response surface analysis method, UPLC-ESI-MS/MS, network pharmacology, molecular docking

## Abstract

*Scrophulariae Radix* is a traditional Chinese medicine used to treat neoplasms in previous publications. Nevertheless, how the *Scrophulariae Radix* chemical constituents treat neoplasm still needs to be clarified. Herein, we combined the response surface method, UPLC-ESI-MS/MS, network pharmacology, molecular docking, and molecular dynamics (MD) simulation to characterize bioactive constituents in *Scrophulariae Radix* and further uncover their potential mechanisms against neoplasm. As a result, the material–liquid ratio was significantly reduced from 100 g/mL to 32 g/mL, and the extraction efficiency was 1.332%, which was close to the predicted value of 1.346% in the response surface method, indicating that the algorithm model had a good fit. Next, a total of 738 compounds, including 161 terpenoids, 144 phenolic acids, 51 alkaloids, 24 flavonoids, 34 saccharides, 32 lignans and coumarins, 45 amino acids and derivatives, 23 organic acids, 134 lipids, 22 nucleotides and derivatives, and 59 other ingredients, were characterized from *Scrophulariae Radix* based on the accurate precursor and product ions, retention time, standards, fragmentation patterns, and previous publications. Subsequently, to screen which constituents were most effective, the network pharmacology was constructed, and 96 active compounds and 488 key neoplasm-related targets were identified, leading to the establishment of the “Drug-Compound-Target” network and PPI network. The top 4 components and targets were selected for the presentation of MD simulation, consisting of cytosporone C, cystomexicone A, mediterraneone, and bestim, with the highest degree related targets carbonic anhydrase 12 (CA12), carbonic anhydrase 2 (CA2), carbonic anhydrase 9 (CA9), and carbonic anhydrase 1 (CA1) being considered as the core compounds and targets. GO pathway analysis was closely related to hormone, protein phosphorylation, and protein kinase activity. KEGG pathway enrichment primarily involved pathways in cancer and the cAMP signaling pathway in cancer. Overall, this integration method provided guiding significance for the exploration of TCM treatment.

## 1 Introduction

Neoplasm has become the predominant health hazard in China and a major threat to human health worldwide (Ma et al., 2019; Wang et al., 2023), and its high incidence and mortality render it challenging to manage effectively (Miao et al., 2023). Despite recent medical advancements, neoplasm patients still face high morbidity and mortality rates due to the limited efficacy of existing treatments. This has drawn significant global research attention. Surgical resection and chemotherapy represent the main modalities of neoplasm treatment (Wang et al., 2018a; Tse et al., 2021). However, surgical treatment remains suitable only in the early stages of some cancers, while chemotherapy often engenders a decline in immune function, drug resistance development, tumor metastasis promotion, and recurrence (Ma et al., 2019). Therefore, a comprehensive approach encompassing multiple targets rather than a singular targeted strategy is the potential trend in the future treatment of tumors (Ma et al., 2019; Miao et al., 2023). Derived from natural substances, traditional Chinese medicine (TCM) exhibits a few side effects; moreover, TCM possesses multiconstituents, multitarget, and multisignaling pathways, thereby enhancing the function of the human immune system while playing a therapeutic role (Gu et al., 2022; Miao et al., 2023) and also has been increasingly used in the development of anti-neoplasm drugs (Qi et al., 2016; Kong et al., 2023).

*Scrophulariae Radix*, a traditional Chinese medicine called “Xuanshen,” is derived from the dried roots of *Scrophularia ningpoensis* Hemsl (Wang et al., 2018b). It is recognized as one of the prominent herbal remedies in Zhejiang Province, referred to as the “eight flavors of Zhejiang” (Zhou et al., 2014). Meanwhile, its abundant pharmacological activity and clinical application value have attracted the attention of scholars worldwide for extensive research and discussion. In addition to its traditional therapeutic effects such as fire pursing, blood cooling, and toxin removal (Lee et al., 2021), numerous clinical trials and pharmacological activity experiments have demonstrated that *Scrophulariae Radix* also possesses anti-angiogenic effect (Wang et al., 2004), anti-inflammatory effect (Díaz et al., 2004), antimicrobial activities (Li et al., 2009), ventricular remodeling activities (Huang et al., 2012), and anti-neurodegenerative disease activities (Kim et al., 2023) as well as the effect of treating neoplasm (Shen et al., 2012; Baradaran et al., 2017; Lewenhofer et al., 2018). Phytochemical investigation revealed that constituents of *Scrophulariae Radix* dominantly consist of iridoid glycosides, phenylpropanoid glycosides, alkaloids, flavonoids, organic acids, and so on (Chen et al., 2008). These various constituents are considered to contribute significantly to the therapeutic effects. Despite several reported phytochemical types of research, the comprehensive chemical profile of *Scrophulariae Radix* remains elusive. Given the complex chemical composition, pharmacological effects, and multiple targets of *Scrophulariae Radix*, it is crucial to investigate its potential pharmacodynamic constituents and mechanisms underlying its therapeutic effects. Therefore, effective methods for structural elucidation and identification of compounds in *Scrophulariae Radix* are essential for discovering novel bioactive constituents.

However, the diversity of the chemical constituents of *Scrophulariae Radix* results in complex interactions between bioactive constituents and multitargets in diseases. It presents a significant challenge in comprehensively excavating potential effective compounds and their action mechanisms (Huang et al., 2021). Currently, the research is focused on characterizing low-content but significant potential bioactive constituents of *Scrophulariae Radix* as a novel, effective treatment option against cancer. Nevertheless, due to the highly heterogeneous and diverse structures of iridoid/phenylpropanoid glycosides, substantial iridoid/phenylpropanoid glycosides that might show as low-abundance/trace species need to be explored. Thus, detecting low-abundance iridoid/phenylpropanoid is challenging due to the ion suppression of co-eluted species. Particularly, the interfering signals arising from the matrix, contaminants, and isomers might compromise the MS/MS spectra of iridoid/phenylpropanoid glycosides, thus hindering the identification of iridoid/phenylpropanoid glycosides. To solve these challenges, we herein developed a response surface analysis procedure before LC-MS analysis of iridoid/phenylpropanoid glycosides. Currently, ultra-performance liquid chromatography coupled with electrospray ionization tandem mass spectrometry (UPLC-ESI-MS) is a powerful qualitative and quantitative analysis technique, which has been developed for rapid and accurate characterization of unknown trace components in TCM due to its high sensitivity, high resolution, and high selectivity (Zhou et al., 2010; Wang et al., 2021). Among various MS types, the tandem quaternary bar linear ion trap mass spectrometry (QTRAP-MS) instrument has the unique advantage of achieving accurate mass measurement, providing the elemental composition of precursor ions and characteristic fragment patterns. This method significantly benefits characterizing and elucidating the unknown trace structural constituents.

Nevertheless, which constituents are the most effective, the potential target, and the action mechanisms are not fully understood, which limits the application of *Scrophulariae Radix*. Thus, the relationship reactions between bioactive constituents in *Scrophulariae Radix* and proteins and genes were also limited. Meanwhile, a conjunction analysis of the biochemical properties of *Scrophulariae Radix* with potential targets and corresponding action mechanisms was necessary. Up to now, network pharmacology has been regarded as an emerging subfield of TCM development according to its integrated analysis of biomedical data with systems medicine (Almuhayawi et al., 2022; Wang and Li, 2022). Via importing biomedical data, the network relationships between bioactive constituents in traditional medicines and corresponding target proteins could be established, consequently unveiling the mechanism of action for synergistic therapeutics in traditional drugs. Therefore, network pharmacology analysis has aided in transitioning the medicines discovery domain from the regular “one target, one drug” mode to the “network target, multiconstituent treatment” method (Li et al., 2022a). In a word, network pharmacology provides a systematic approach to elucidate intricate interactions among TCM, chemical compounds, targets, diseases, and pathways, which has been considered to be a reliable method to predict the bioactive mechanism of TCM treatments for diseases based on advancements in systems bioinformatics (Lu et al., 2021b). Notably, comprehending the intricate landscape of network pharmacology is pivotal in determining candidate drugs (Noor et al., 2022).

Despite network pharmacology and molecular docking providing an assessment of constituent suitability in the protein bioactive site, they solely offer information limited to the protein active site. Thus, to estimate the compound–protein target system further, the application of binding conformation information has already become more widespread and requires the application of molecular dynamics (MD) simulations, as well as their related binding energy measurements. The utilization of MD simulations promotes a comprehensive exploration of the dynamic properties exhibited by docked complexes and fluctuations in the energy action. Such insights serve a crucial role in confirming the stability of the complex and determining feasible structural transformations in the protein activated by ligand interaction. In short, the evaluation arises from molecular docking, and MD simulations underscore a considerable binding affinity in the interactions involving active compounds and proteins.

**Figure 1:**
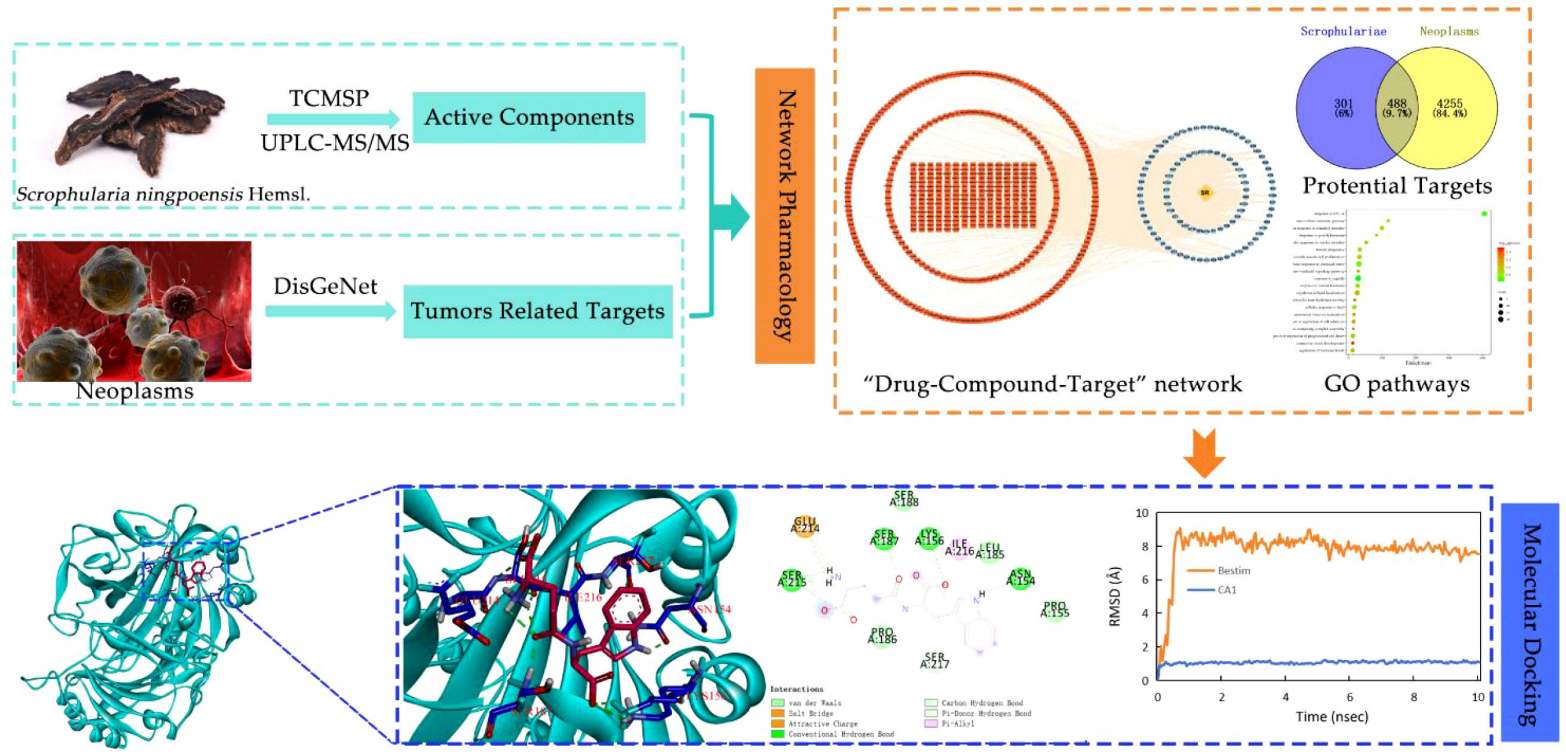
The study workflow includes identification of active ingredients, validation and prediction of network pharmacology, and molecular docking, to elucidate the mechanism underlying the antitumor effects of *Scrophulariae Radix*.

In the present study, to extract more trace amounts of active constituents, we first optimized the sample preparation conditions by adopting the response surface analysis method, and then a rapid and sensitive UPLC ESI-MS/MS method was established to elucidate and characterize major complex chemical constituents of *Scrophulariae Radix*. Next, these chemical constituents were explored by network pharmacology, molecular docking, and MD simulation to investigate the active constituents of *Scrophulariae Radix* and explore their potential target proteins, to elucidate the underlying mechanisms of its anti-neoplasm activities. Hence, this study presents certain guiding significance for clinical application and exploration mechanisms of action of *Scrophulariae Radix* in the treatment of neoplasm, as well as provides ideas for further pharmacological experimental verification and development of new antitumor drugs. The study workflow is shown in **Figure 1**.

## 2 Materials and methods

### 2.1 Chemicals and reagents

A total of 13 reference substances of angoroside C (CAS:115909-22-3), harpagoside (CAS:19210-12-9), cinnamic acid (CAS:140-10-3), harpagide (CAS:6926-8-5), aucubin (CAS:479-98-1), catalpol (CAS:2415-24-9), verbascoside (CAS:61276-17-3), p-coumaric acid (CAS:501-98-4), ferulic acid (CAS:1135-24-6), caffeic acid (CAS:331-39-5), fumaric acid (CAS:110-17-8), geniposide (CAS:24512-63-8), and 5-hydroxymethylfurfural (CAS:67-47-0) were purchased from Shanghai Yuanye Bio-Technology Co. Ltd. (Shanghai, China). The purity of each standard was above 98%. HPLC-grade methanol and acetonitrile were purchased from Tedia (Tedia Way, Fairfield, USA). Ultrapure water was prepared using a Milli-Q purifying system (Millipore, Bedford, MA, USA). Formic acid (HPLC grade) was supplied by Nanjing Chemical Reagent Co., Ltd. (Nanjing, China). All the other chemicals were of analytical grade.

### 2.2 Plant material

The fresh roots of *S. ningpoensis* were investigated and collected from Badong County, Enshi City, Hubei Province, P.R. China. These herbal samples were authenticated by Professor Lu-Ping Qin (School of Pharmaceutical Sciences, Zhejiang Chinese Medical University, Hangzhou City, Zhejiang Province, P.R. China). Meanwhile, the samples were deposited in the School of Pharmaceutical Sciences, Zhejiang Chinese Medical University, with the code 3307023100508159SY.

### 2.3 Optimization of sample preparation

Using vacuum freeze-drying technology, the biological samples were placed in a lyophilizer (Scientz-100F) and then ground (30 Hz, 1.5 min) to a powder form using a grinder (MM 400, Retsch). Next, 50 mg of the sample powder was weighed using an electronic balance (MS105DM) and then added with 1,200 μL of −20°C precooled 70% methanolic aqueous internal standard extract (less than 50 mg added at the rate of 1,200 μL extractant per 50 mg sample). Then, the samples were vortexed six times, once every 30 min for 30 s. After centrifugation (rotation speed 12,000 rpm, 3 min), the supernatant was aspirated, and the sample was filtered through a microporous membrane (0.22 μm pore size) and stored in the injection vial for UPLC-MS/MS analysis.

### 2.4 Preparation of reference substance solutions

Individual stock solutions of standards (1 mg/mL) were accurately weighed and prepared by dissolving in methanol. Then, the solutions of 13 reference substances (angoroside C, harpagoside, cinnamic acid, harpagide, aucubin, catalpol, verbascoside, p-coumaric acid, ferulic acid, caffeic acid, fumaric acid, geniposide, 5-hydroxymethylfurfural) were mixed, and a stock reference solution was obtained. All stock and working reference solutions were filtered through a 0.22-µm filter and stored at −20°C until they were used for UPLC-MS/MS analysis.

### 2.5 UPLC-MS conditions

Each sample was analyzed using a Waters ACQUITY UPLC H-Class system (Waters, Milford, MA, USA), equipped with an ACQUITY UPLC BEH C_18_ column (100 mm × 2.1 mm with 1.7 µm particle size, Waters). The mobile phase consisted of acetonitrile (solvent A) and water containing 0.1% formic acid (v/v) (solvent B) at a flow rate of 0.2 mL/min. The gradient elution was as follows: 5% A at 0–2 min; 5%–23% A at 2–7 min; 23%–24% A at 7–9 min; 24%–35% A at 9–11 min; 35%–95% A at 11–14 min; and decreasing from 95% to 5% A at 14–15 min. The column temperature was maintained at 35°C and the sample injection volume was 2 μL. The effluent was alternatively connected to an ESI-triple quadrupole-linear ion trap (QTRAP)-MS.

The UPLC system was coupled to a Biosystems QTrap 6500 mass spectrometer from AB Sciex (https://sciex.com.cn/, Warrington, UK) equipped with an electrospray ionization (ESI) turbo V ion spray source operated in both positive and negative modes for detection with the mass range of *m*/*z* 100–1,200. The ESI source operation parameters were as follows: source temperature, 550°C; ion spray voltage (IS), 5,500 V (positive ion mode)/−4,500 V (negative ion mode); ion source gas I (GSI), gas II (GSII), and curtain gas (CUR) were set at 50, 60, and 25 psi, respectively; the collision-activated dissociation (CAD) was high. Full scans were acquired as multiple reaction monitoring (MRM) experiments with collision gas (nitrogen) set to medium. Declustering potential (DP) and collision energy (CE) for individual MRM transitions were done with further DP and CE optimization. A specific set of MRM transitions was monitored for each period according to the metabolites eluted within this period.

The data were processed using the software Analyst 1.6.3. Based on the self-built MWDB (Metware database), the secondary spectrum information was used for qualitative analysis, and the quantitative analysis was performed using MRM. After screening out the characteristic ions of each substance by triple quadrupole mass spectrometry, the characteristic ion signal strength was obtained in the detector. Furthermore, the fragmentation patterns of reference standards were used to characterize components that possess similar skeleton structures or fragment ions based on the principle of structural similarity.

### 2.6 Response surface design

The effects of methanol volume fraction, solid–liquid ratio, soaking time factor, and ultrasonic time on the extraction of six components (fumaric acid, caffeic acid, p-coumaric acid, ferulic acid, angloside C, and harpagoside) were studied. After analyzing the single-factor test results, the Box–Behnken design with three factors and three levels was adopted for the surface analysis test. When other factors were fixed at the optimal value, the methanol volume fraction and the feed liquid were selected. The ratio and soaking time of the six components of *Scrophulariae Radix* were the main factors affecting the extraction rate. The total extraction rate was the response value; the response surface method was used to analyze the test; and the response surface test results were examined through differential analysis.

### 2.7 Network pharmacology analysis

#### 2.7.1 Target collection and prediction

Screening of active compounds and related targets: Firstly, to generate a more comprehensive collection, all the identified components based on UPLC-MS analysis were transformed into canonical Simplified Molecular Input Line Entry System (SMILES) through the PubChem database (https://pubchem.ncbi.nlm.nih.gov/) (Wang et al., 2012; Kim et al., 2021), which was most recently updated in 2019. Nevertheless, numerous compounds were not found in the PubChem database. The details of these structures were obtained from the Traditional Chinese Medicine Systems Pharmacology (TCMSP) database (https://old.tcmsp-e.com/tcmsp.php) (Ru et al., 2014). Molecules with better pharmacokinetic properties were screened with the help of Lipinski’s rule (Lipinski et al., 2001), with the molecular weight (MW) not exceeding 500, the XLogP smaller than 5, the H-bond donors less than 5, and the H-bond acceptors less than 10. Their structures were shown by ChemDraw 20.0. Next, their structures were imported into the BindingDB (http://bindingdb.org/bind/index.jsp), DrugBank (https://go.drugbank.com/), STITCH (http://stitch.embl.de/), and SwissTargetPrediction (https://swisstargetprediction.ch/) (Gfeller et al., 2014; Daina et al., 2019) databases to achieve SMILES, and the canonical SMILES was uploaded into the SwissTargetPrediction database in “*Homo sapiens*” species to predict potential targets of chemical components with a screening threshold >0.1.

Predicting targets of neoplasm: We used SwissTargetPrediction to indicate targets and obtained “neoplasm”-related genes on DisGeNET (https://www.disgenet.com) (Piñero et al., 2019), DrugBank (https://go.drugbank.com/), OMIM (https://www.omim.org/), and GeneCards (https://www.genecards.org/). Only the protein-coding gene targets were selected for the subsequent analysis. Finally, Venny 2.1.0 (https://bioinfogp.cnb.csic.es/tools/venny/index.html) was used to draw the Venn diagram of intersection genes, and the protein–protein interaction (PPI) network map of intersection genes was obtained through the String website (https://cn.string-db.org/).

#### 2.7.2 Network construction and analysis

##### 2.7.2.1 Targets overlapping between components and neoplasm

The overlapping targets and active ingredients were imported into Cytoscape ver. 3.10.0 to construct the Drug-Compound-Target network, and the “Network Analyzer” function was used to analyze the topological properties of the network. Firstly, the “Drug-Compound-Target” network of *Scrophulariae Radix* for treating neoplasm was constructed by Cytoscape 3.10.0 (Shannon et al., 2003). Nodes in the network represent compounds, targets, and pathways, while edges represent interactions between two nodes. To identify critical components and target genes, the CentiScaPe 2.2 plug-in for Cytoscape was used for calculating the betweenness, closeness, and degree of each node.

##### 2.7.2.2 Protein–protein interaction network

The protein–protein interaction network was analyzed and visualized using STRING ver. 12.0 (https://string-db.org/). To further investigate the mechanism of action of *Scrophulariae Radix* in the treatment of neoplasm, the intersection targets were imported into STRING. The minimum required interaction score was set to 0.7 and the protein–interaction relationships for *Homo sapiens* were selected for continued analysis. Then, the PPI network was established by importing the node and the combined score into Cytoscape. The targets without interaction were removed. Finally, the core targets were screened by applying the CentiScaPe 2.2 plug-in in Cytoscape.

##### 2.7.2.3 GO and KEGG pathway enrichment analysis

Gene Ontology (GO) enrichment analysis was performed by running the “clusterProfiler” package in R Foundation for Statistical Computing. The intersection of previously predicted targets and “neoplasm”-related genes was imported into Metascape (https://www.metascape.org) to obtain GO information, in which modules of biological processes (BP), molecular function (MF), and cellular component (CC) were selected, and significant biological annotations (*p* < 0.05) were chosen for analysis (Ashburner et al., 2000). Meanwhile, KEGG pathway enrichment analysis was carried out to investigate the major signaling pathways involved in the antitumor effects of *Scrophulariae Radix* (Kanehisa and Goto, 2000; Kanehisa et al., 2017). Finally, the enriched results were analyzed employing the “SRplot” package in R to make the plot (Tang et al., 2023).

##### 2.7.2.4 Drug-Compound-Target network

To further clarify the relationship between bioactive ingredients and target genes, the Drug-Compound-Target network relationship was established by intersecting significant targets with the Drug-Compound network. These network results were visualized in Cytoscape.

##### 2.7.2.5 Molecular docking

Molecular docking could be helpful to validate critical targets in network pharmacology. Firstly, the structure of the core compounds was obtained through the PubChem database (https://pubchem.ncbi.nlm.nih.gov/) (Wang et al., 2012; Kim et al., 2021) and mapped using ChemDraw 20.0, which was saved and exported in “sdf” format. Meanwhile, the 3D crystal structure of the core ligand protein was obtained in the RCSB Protein Data Bank (PDB) database (https://www.rcsb.org/), and then the ligand protein was dehydrated, charge-edited, and hydrogenated in the AutoDock software, then visualization was performed through Discovery Studio, and the modified target proteins (Clean protein in the Prepare Protein of Macromolecules to supplement the possible missing parts of protein structure) and important chemical component were exported as PDB files.

##### 2.7.2.6 Molecular dynamics simulations

In order to further verify the results of molecular docking, 10-ns molecular dynamics simulation of the foregoing four ligand–receptor conformation was performed using Desmond version 2.3 (Maestro version 13.4) (Sankar et al., 2022). The complexes were solvated in orthorhombic boxes filled with TIP3P water molecules (Jorgensen et al., 1983), maintaining a 10-Å buffer distance from the box boundaries. The ion placement was neutralized by adding Cl^−^ ions randomly and parametrized applying the OPLS4 force field (Lu et al., 2021a). The simulations were carried out in the isothermal–isobaric (NPT) ensemble at temperature of 300 K and pressure of 1.013 bar for a 2-fs time step. The trajectory and energy were logged at intervals of 50 ps and 1.2 ps, respectively. The root mean square deviation (RMSD) and root mean square fluctuations (RMSF), the number of H-bonds, the radius of gyration (Rg), and energy were calculated by simulation interactions diagram in Maestro version 13.4.

## 3 Results

### 3.1 Optimization of sample preparation by response surface analysis method

**Figure 2:**
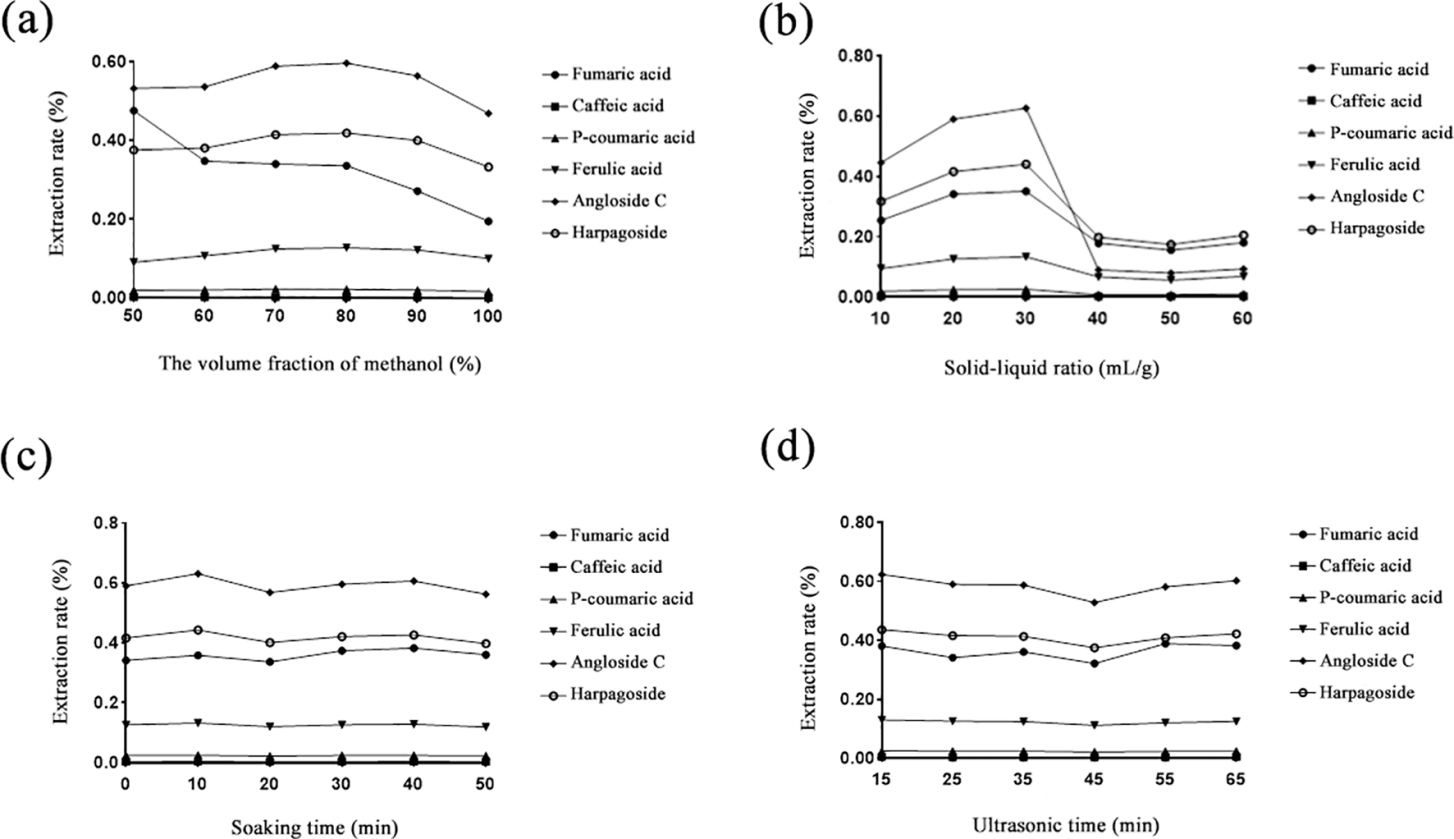
Effect of six reference standards of *Scrophulariae Radix* extraction rate. **(a)** Methanol volume fraction; **(b)** solid–liquid phase; **(c)** soaking time; **(d)** ultrasound time.

To extract more active ingredients, sample extract preparation was optimized by the response surface analysis method using six reference standards (fumaric acid, caffeic acid, p-coumaric acid, ferulic acid, angloside C, and harpagoside). Firstly, based on the single-factor test, the extracting parameters were screened by the larger influence range. Hence, the experimental optimized parameters were carried out with the response surface analysis method, including methanol volume fraction (v/v, 50%~100%), solid–liquid phase (10~60 mL/g), soaking time (0~50 min), and ultrasound time (15–65 min) shown in **Figure 2**.

**Figure 3:**
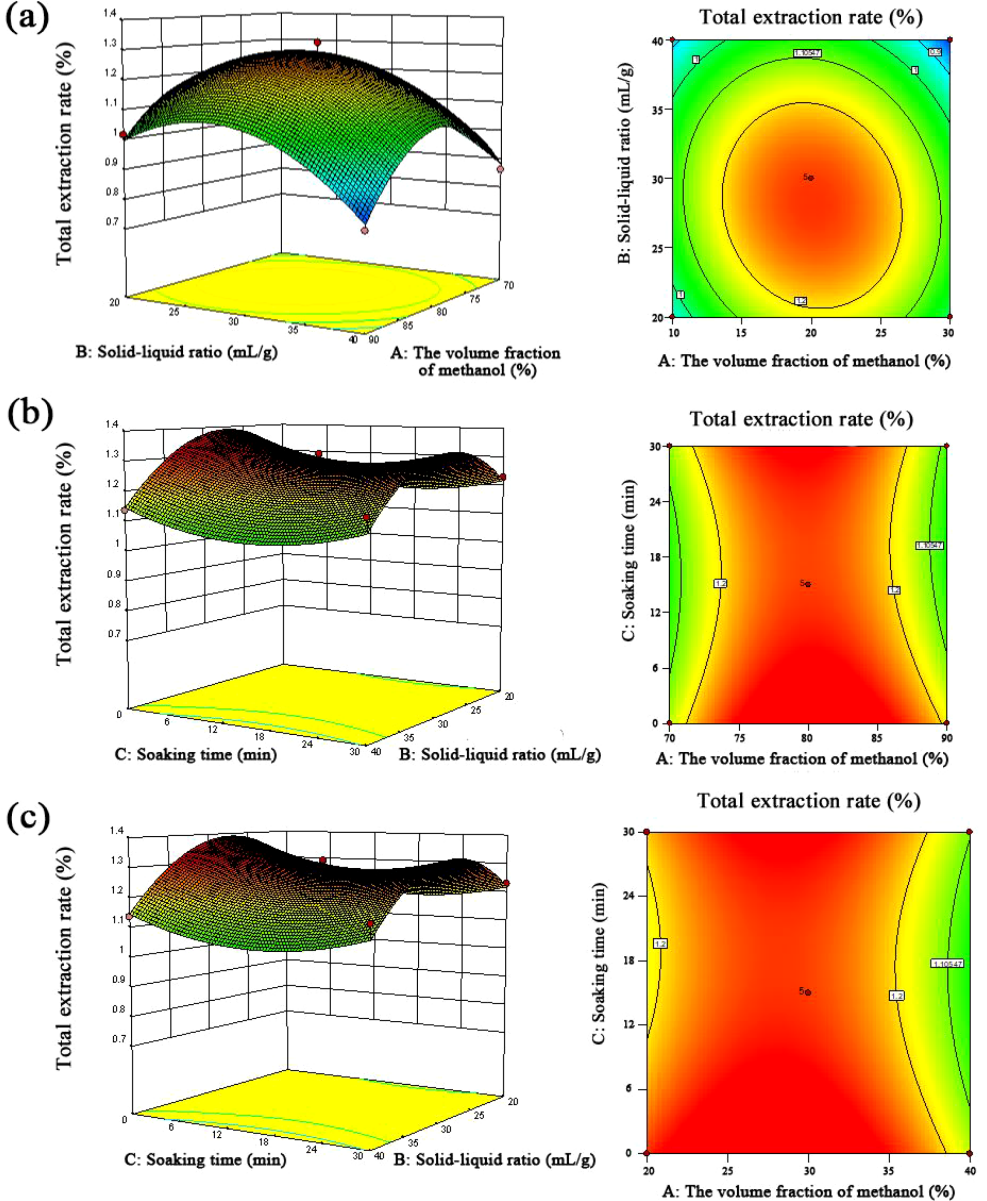
The effects of the interaction on the extraction rates of six components of *Scrophulariae Radix*. **(a)** Methanol volume fraction and solid–liquid ratio; **(b)** methanol volume fraction and soaking time; **(c)** solid–liquid ratio and soaking time.

Secondly, the response surface analysis test results were analyzed by Design-Expert 10.0.7 software, and the total extraction rate of the six components was used as the response value. Thirdly, after the multidirectional fitting regression of the experimental data, the regression equation was obtained as follows: *Y* = 14.57766 + 0.35574 + 0.11804007895893 B − 0.00157392511749912 * 10^−3^ C − 3.45927415898910 * 10^−4^ AB − 1.47410057892483 * 10^−4^ AC + 6.43457676683007 * 10^−5^ BC − 2.14772329141351 * 10^−3^ A^2^ − 1.62794250022502 * 10^−3^ B^2^ + 3.20350600800245 * 10^−4^ C^2^, analysis of variance for fitted equations. Next, a *p*-value <0.01 of the regression model indicates that the regression model had high significance. Then, *p* = 0.1409 > 0.05 of the missing term stated that there was no significant difference in the missing term, and the equation had a good fit, which could be used as the basis for analysis and prediction of the extraction rate of *Scrophulariae Radix.* Fourthly, the interactive analysis indicated that the shape of the response surface analysis model was positively correlated with the sensitivity of the response value. Meanwhile, the steeper the slope of the response surface, the more sensitive the response value. The effects of the interaction on the extraction rates of the six components are shown in **Figure 3**. It can be seen from the interaction model data that AB interaction was stronger than that of AC and BC, indicating that the interaction effect of methanol volume fraction and solid–liquid ratio on the extraction rate of the six components of *Gentiana* was more obvious. The order of interaction terms from strong to weak was AB > AC > BC, which was the same as the results of the significance test. Fifthly, for optimal process reliability verification results, Design-Expert 10.0.7 software was used to obtain the optimal parameters under the conditions of a methanol volume fraction of 79.821%, solid–liquid ratio of 32.486 mL/g, and soaking time of 0.745 min. On this basis, combined with the actual operability, the parameters were changed as follows: methanol volume fraction 80%, solid–liquid ratio 32 mL/g, and soaking time 0 min. The average value of the total extraction rate was 1.332%, and the relative deviation from the predicted value of 1.346% was −1.049%, which showed that the results obtained by this method were reliable.

Finally, the optimal conditions for the comprehensive extraction of reference standards were obtained as follows. The optimum extraction parameters were 80% methanol, 32 g/mL solid-to-liquid ratio, 0 min soaking time, and ultrasound time of 15 min. Hence, the experimental extraction efficiency of the six reference standards was 1.332%. Then, the extraction of *Scrophulariae Radix* was improved, and the material–liquid ratio was reduced from 100 g/mL to 32 g/mL.

### 3.2 Structural characterization and identification of major chemical constituents of *Scrophulariae Radix* through UPLC-ESI-MS combined with cleavage pathways

**Figure 4:**
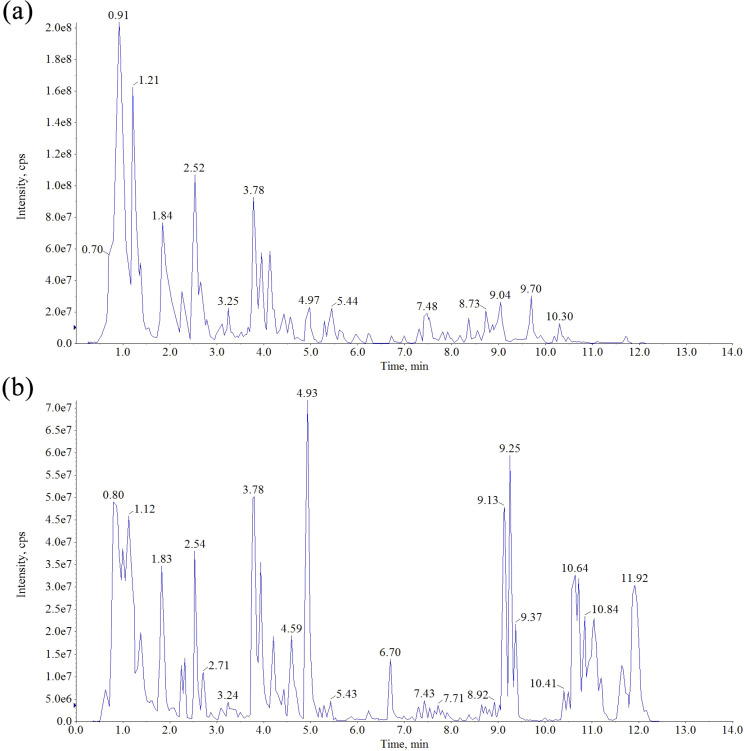
Total ion chromatogram in the positive ion mode **(a)** and negative ion mode **(b)** for the extract of *Scrophulariae Radix*.

The chemical constituents of *Scrophulariae Radix* were detected and elucidated using UPLC-MS in both positive and negative modes to obtain as much information as possible. The total ion chromatograms (TICs) of these ingredients in positive and negative ion modes are shown in **Figure 4**. Moreover, 13 ingredients in *Scrophulariae Radix* were unambiguously identified using comparison with their standards. According to multistage mass spectrometry characteristic fragment ions, some new features of the fragmentation pattern of iridoid and phenylpropanoid compounds were summarized as follows.

#### 3.2.1 Structural identification and fragmentation pattern of iridoid glycosides

The phenylpropyl structural iridoid glycosides were predominated in *Scrophulariae Radix*. According to the current UPLC-MS experimental results, the iridoid glycosides of *Scrophulariae Radix* contained three subtypes, namely, cyclopentane, cyclopentene, and epoxy cyclopentane (Wu et al., 2010). Under the MS collision energy, the phenylpropyl structural unit (ester bond break) was firstly the neutral loss, and then it was found that the neutral loss of a glucoside group (glucoside bond break) was a typical fragmentation for iridoid glycosides in *Scrophulariae Radix*. Next, the neutral loss of an H_2_O group was further lost due to the presence of a hydroxyl group on the iridoid aglycones. In addition, the iridoid group may undergo ring opening and further cleavage. Next, the fragmentation pattern of different types of structures was analyzed and summarized.

**Figure 5:**
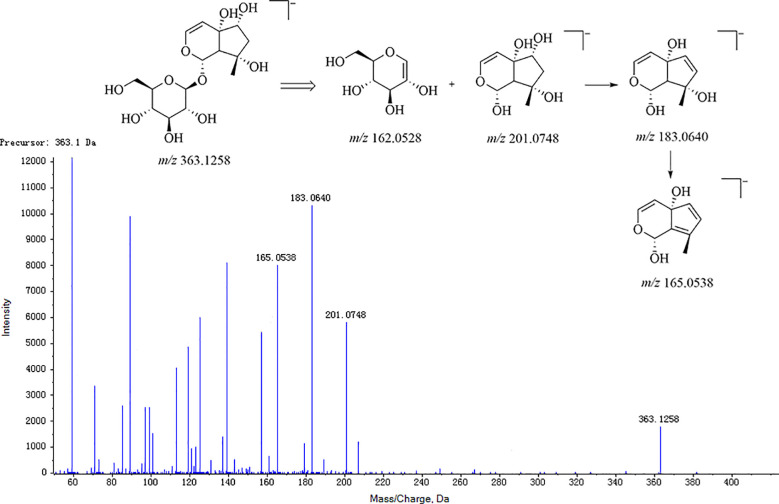
MS fragmentation pattern of cyclopentane-type iridoid glycosides (e.g., harpagide).

Firstly, for the fragmentation pattern of cyclopentane-type iridoid glycosides, the precursor ions were *m*/*z* 201.08 [M-H]^−^ by the break of the ester bond and glycoside bond, among which the characteristic fragments were *m*/*z* 183.07 [M-H_2_O-H]^−^ and *m*/*z* 165.06 [M-2H_2_O-H]^−^. Taking the harpagide structural elucidation as an example, the precursor ions were *m*/*z* 363.1258 [M-H]^−^ in the MS, and the fragment ions were respectively *m*/*z* 245, 201, 183, 179, 165, and 161, wherein the characteristic fragment ions were *m*/*z* 183 and 165, where their feature ion was derived from the precursor ion *m*/*z* 363.1258 through the sequential loss of a glucose group (C_6_H_12_O_6_) and the neutral loss of an H_2_O group successively. Meanwhile, ion *m*/*z* 363.1258 fractured the glucoside bond and lost the precursor nuclear fragment C_9_H_12_O_4_ (*m*/*z* 184) to generate ion *m*/*z* 179 [C_6_H_12_O_6_-H]^−^ and ion *m*/*z* 183 [C_6_H_12_O_6_-H_2_O-H]^−^. Next, the precursor ions were neutral loss of *m*/*z* 162 dehydrated glucose (C_6_H_10_O_5_), and an H_2_O group produced fragment ions *m*/*z* 201 [M-Glc-H]^−^ and *m*/*z* 345 [M-H_2_O-H]^−^, respectively. The harpagide MS/MS and cleavage pathway is shown in **Figure 5**. This compound was unequivocally identified as harpagide through comparison with an authentic standard.

Similarly, for another example of harpagoside, the precursor ion was *m*/*z* 493.1589 [M-H]^−^, and their characterized fragment ions *m*/*z* 345 and *m*/*z* 327 were obtained by the precursor ion losing a group of cinnamic acid successively (C_9_H_8_O_2_) and a group of H_2_O. Moreover, ions *m*/*z* 179 [C_6_H_12_O_6_-H]^−^ and *m*/*z* 161 [C_6_H_12_O_6_-H_2_O-H]^−^ were regarded as characteristic fragment ions of glucose. This compound was unequivocally identified as harpagoside through comparison with an authentic standard.

Therefore, most of the iridoid compounds were glucosides, and then the common phenylpropionyl substituents were cinnamyl, p-coumaryl, caffeoyl, feruloyl, etc. Based on the analysis of the above cleavage rule, the iridoid glycosides in MS that displayed the precursor ion peaks [M-H]^−^ showed that H_2_O, glucose (*m*/*z* 162), cinnamic acid (*m*/*z* 148), p-coumaric acid (*m*/*z* 164), caffeic acid (*m*/*z* 180), ferulic acid (*m*/*z* 194), and other neutral fragment ions were easily fractured. Moreover, the characteristic fragment ions *m*/*z* 203 and *m*/*z* 185 of glucose were detected using secondary mass spectrometry.

For cyclopentene-type iridoid glycosides, ion *m*/*z* 183.07 [M-H]^−^ was produced by the parent ion after the ester bond and glycoside bond break, and its characteristic fragment ions were *m*/*z* 165.06 [M-H_2_O-H]^−^ and *m*/*z* 135.06 [M-H_2_O-CH_2_O-H]^−^ as shown in **Figure 5**.

According to this rule, the structures of cyclopentane-type iridoid glycosides harpagide, 6′-O-cinnamoyl harpagide, and 8-O-(p-coumaroyl) harpagide, etc. were detected. Moreover, cyclopentene-type iridoid glycoside 6′-O-glucosylaucubin was also analyzed. These observations were consistent with previous reports. Detailed data are shown in **Supplementary Table 1**.

#### 3.2.2 Structural identification and fragmentation pattern of phenylpropanoid glycosides

Another major group of bioactive ingredients in *Scrophulariae Radix* was the phenylpropanoid glycosides. Generally, a phenylpropanoid glycoside consists of a phenylpropanol group and a saccharide unit. The composition of this type of ingredient differs in the substituted phenylpropionic acid and other saccharide units bonding to the phenpropanol and saccharide groups. In this study, the basic skeleton of phenylpropanoid glycosides was mainly characterized by α, β unsaturated phenyl propionic acid [including cinnamic acid (M *=* 148), ferulic acid (M *=* 194), caffeic acid (M = 180), coumaric acid (M = 164)] and the saccharic fraction [including glucose (M = 180), rhamnose (M = 164), arabinose (M = 150)]. Moreover, based on the corresponding collision energy, the phenylpropanoid glycosides often cause the neutral loss of phenylpropanoid structural groups, followed by rhamnosyl (M = 146) and finally arabinose (M = 132).

**Figure 6:**
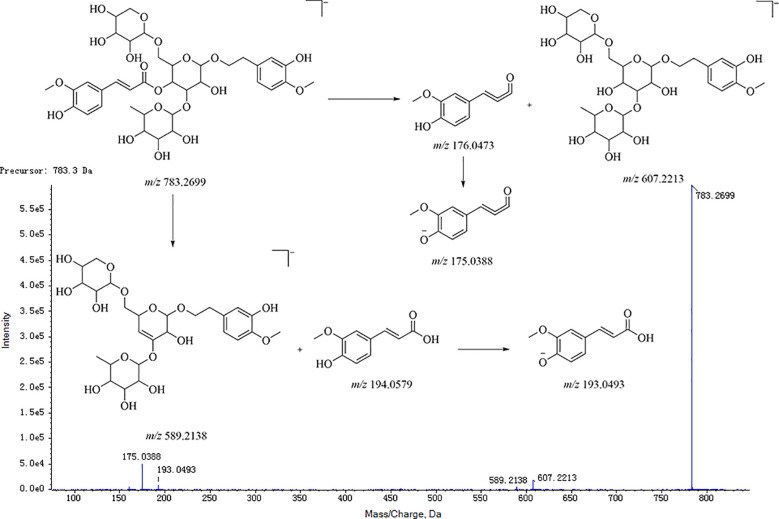
MS fragmentation pattern of phenylpropanoid glycosides (e.g., angoroside C).

Taking the angloside C structural elucidation as an example, the precursor ions of angloside C in MS was *m*/*z* 783.2699 [M-H]^−^. Their precursor ions produced characteristic fragment ions *m*/*z* 607 and *m*/*z* 589 in MS/MS successively with the neutral loss of arabinose (*m*/*z* 132), rhamnose (*m*/*z* 146), and ferulic acid (*m*/*z* 194). Moreover, fragment ions *m*/*z* 505 [M-Rha-Ara-H]^−^, *m*/*z* 457 [M-Ara-ferulic acid-H]^−^, *m*/*z* 443 [M-Rha-ferulic acid-H]^−^, and *m*/*z* 311 [M-Rha-ferulic acid-Ara-H]^−^ could also be detected. The secondary mass spectrum and cleavage pathway of angloside C is shown in **Figure 6**. This compound was unequivocally identified as angloside C through comparison with an authentic standard.

According to the above multistage cleavage pathway analysis, the phenylpropanoid compounds in the MS are mainly shown by the precursor ion peak [M-H]^−^, which enabled the easy breakage of the phenylpropanoid structural fragments (such as cinnamic acid, p-coumaric acid, caffeic acid, and ferulic acid) and the neutral loss of fragments (such as rhamnose and arabinose) and the H_2_O group as well.

#### 3.2.3 Structural identification of other classification compounds

**Figure 7:**
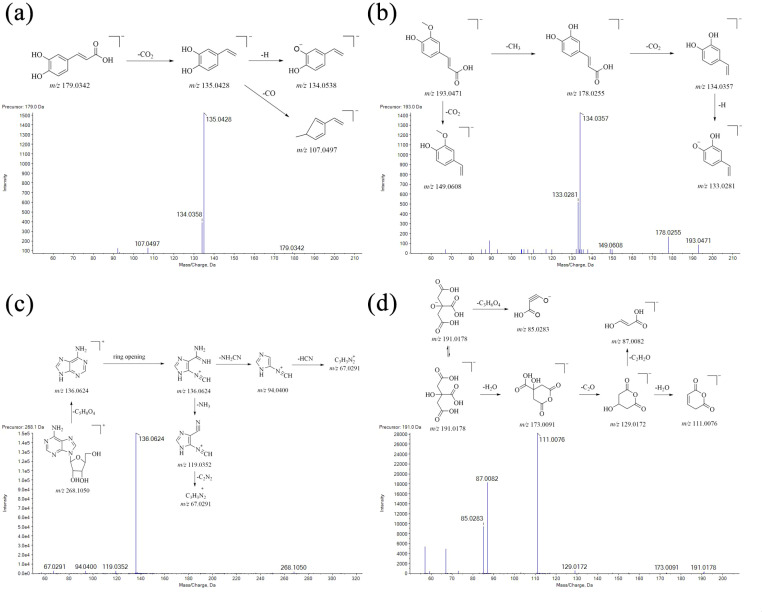
Fragmentation pathways of organic acids in the MS/MS. **(a)** Caffeic acid; **(b)** ferulid acid; **(c)** adenosine; and **(d)** citric acid.

Excluding iridoid glycosides and phenylpropyl glycosides, other classification compounds, such as phenolic acids (caffeic acid and ferulic acid), nucleotides and derivatives (adenosine), and organic acids (citric acid), were detected from *Scrophulariae Radix*. Their MS/MS fragmentation pathways were further elucidated, as shown in **Figure 7**.

Firstly, a dominant protonated ion of caffeic acid was *m*/*z* 179 [M-H]^−^ in the negative ion mode. The base peak at *m*/*z* 135 was formed by the neutral loss of one CO_2_ molecule from the protonated ion. Subsequently, another major fragment ion at *m*/*z* 117 and *m*/*z* 107 was produced by the successive loss of one H_2_O group and one CO group from ion *m*/*z* 179, and then the fragmentation pathway is shown in **Figure 7a**.

Secondly, the precursor ion of ferulic acid was *m*/*z* 193.0506 [M-H]^−^ (C_10_H_11_O_4_), and its characteristic fragment ion *m*/*z* 178 and *m*/*z* 149 was formed by the neutral loss of one CH_3_ group and one CO_2_ group from the protonated ion, respectively. Another major fragment ion at *m*/*z* 134 was produced by the successive loss of one CO_2_ group from *m*/*z* 178, and then the fragmentation pathway is shown in **Figure 7b**.

Thirdly, upon collision induction, a dominant protonated ion of adenosine was *m*/*z* 267.0968 [M-H]^−^. The n-glucoside bond in this protonated ion was easily cleaved, resulting in a neutral loss of one ribose group (*m*/*z* 132) to form a group-produced ion *m*/*z* 136. Subsequently, another major fragment ion at *m*/*z* 119, *m*/*z* 94, and *m*/*z* 67 was produced by the successive loss of one -NH_2_, -NH_2_CN, and HCN group from ion *m*/*z* 136, and then the fragmentation pathway is shown in **Figure 7c**.

Finally, as for citric acid, its protonated ion at *m*/*z* 191 [M-H]^−^ was observed in a negative ion mode. Its characteristic fragment ions *m*/*z* 85 and *m*/*z* 173 were formed by the neutral loss of one C_3_H_6_O_4_ group and one H_2_O group from the protonated ion, respectively. Subsequently, another major fragment ion at *m*/*z* 129, *m*/*z* 111, and *m*/*z* 87 was produced by the successive loss of one -CO_2_, H_2_O, and C_2_H_2_O group from precursor ion *m*/*z* 173, and then the fragmentation pathway is shown in **Figure 7d**.

**Figure 8:**
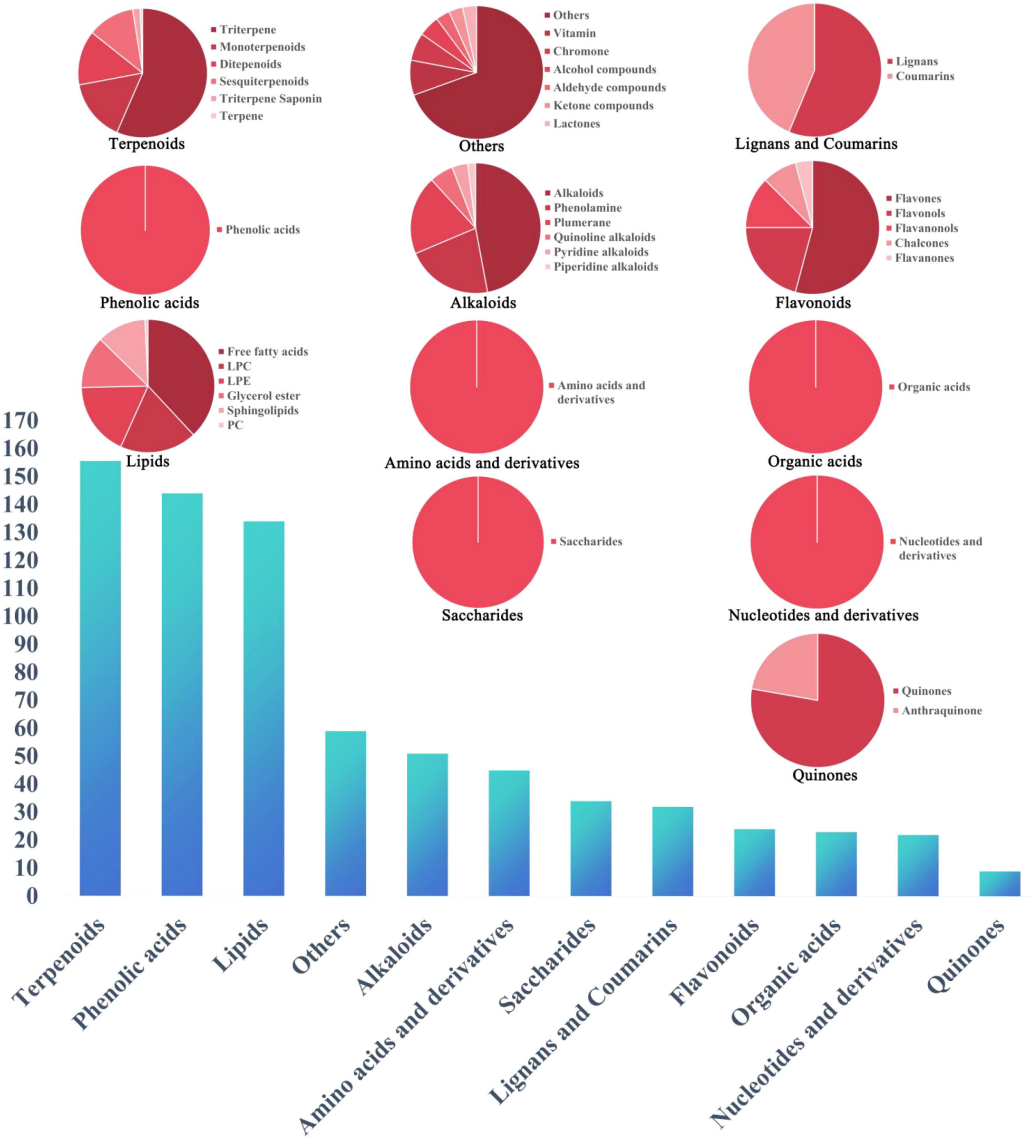
The classification results of 738 compounds in *Scrophulariae Radix* identified by UPLC-ESI-MS/MS.

**Figure 9:**
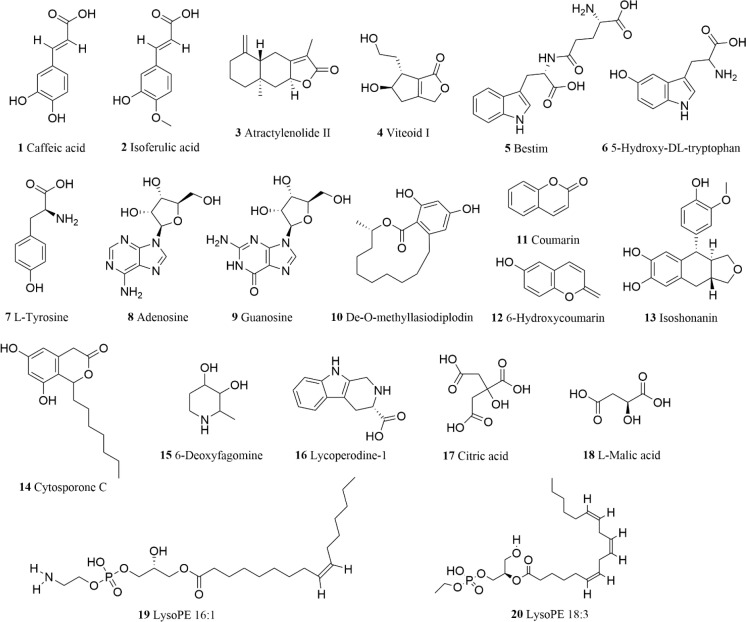
Representative chemical structures of 20 compounds from the extract of *Scrophulariae Radix*.

Based on the above summary, a total of 738 constituents were identified from *Scrophulariae Radix* as shown in **Figure 8**. According to accurate precursor and product ions and comparing their fragmentation patterns with the corresponding reference standards and previous publications, there were 161 terpenoids (including iridoid glycosides), 144 phenolic acids (including phenylpropanoid), 51 alkaloids, 24 flavonoids, 34 saccharides, 32 lignans and coumarins, 45 amino acids and derivatives, 23 organic acids, 134 lipids, 22 nucleotides and derivatives, and 59 other ingredients being presented in **Supplementary Table 1**, among which 20 representative compounds are shown in **Table 1** and their chemical structures are displayed in **Figure 9**.

**Table 1:** Representative metabolites from *Scrophulariae Radix* exacted using UPLC-ESI-MS/MS.

No.	RT (min)	Measured mass (*m*/*z*)	Theoretical mass (*m*/*z*)	Error (ppm)	Formula	Ionization model	Compounds	Classification	CE	MS^2^
1	3.40	179.0336	179.0345	−5.026964077	C_9_H_8_O_4_	[M-H]^−^	Caffeic acid	Phenolic acids	−20.00	135.0466; 134.0380; 107.0495; 117.0352; 79.0553
2	4.03	193.0506	193.0501	2.590001248	C_10_H_10_O_4_	[M-H]^−^	Isoferulic acid	Phenolic acids	−20.00	134.0403; 178.0299; 133.0320; 137.0241; 149.0520
3	8.50	233.1537	233.1541	−1.715603543	C_15_H_20_O_2_	[M+H]^+^	Atractylenolide II	Terpenoids	30.00	187.1444; 159.0853; 131.0832; 215.1397; 151.0724
4	1.40	185.0808	185.0814	−3.241816844	C_9_H_12_O_4_	[M+H]^+^	Viteoid I	Terpenoids	30.00	77.0384;93.0696;91.0541;105.0691;79.0552
5	3.10	334.1406	334.1403	0.897826452	C_16_H_19_N_3_O_5_	[M+H]^+^	Bestim	Amino acids and derivatives	30.00	188.0708; 46.0601; 159.0909; 225.1022; 118.0651
6	2.10	221.0924	221.0926	−0.904598345	C_11_H_12_N_2_O_3_	[M+H]^+^	5-Hydroxy-DL-tryptophan	Amino acids and derivatives	30.00	130.0644; 158.0600; 132.0441; 175.0854; 157.0750
7	1.10	182.0814	182.0817	−1.647612034	C_9_H_11_NO_3_	[M+H]^+^	L-Tyrosine	Amino acids and derivatives	20.00	119.0488; 146.0114; 136.0751; 91.0538; 123.0459
8	1.90	268.1051	268.1046	1.864943757	C_10_H_13_N_5_O_4_	[M+H]^+^	Adenosine	Nucleotides and derivatives	30.00	136.0623; 119.0355
9	1.50	284.0999	284.0995	1.407957423	C_10_H_13_N_5_O_5_	[M+H]^+^	Guanosine	Nucleotides and derivatives	40.00	152.0574; 135.0306; 110.0336; 134.0453; 109.0509
10	9.60	279.1597	279.1596	0.358218023	C_16_H_22_O_4_	[M+H]^+^	De-O-methyllasiodiplodin	Quinones	30.00	149.0248; 121.0301; 57.0707; 65.0393; 93.0337
11	3.30	147.0442	147.0446	−2.720263104	C_9_H_6_O_2_	[M+H]^+^	Coumarin	Lignans and coumarins	30.00	91.0557; 65.0393; 119.0506; 105.0734; 115.0556
12	4.00	163.0389	163.0395	−3.680089794	C_9_H_6_O_3_	[M+H]^+^	6-Hydroxycoumarin	Lignans and coumarins	30.00	89.0419; 117.0378; 77.0416; 63.0242; 135.0446
13	9.20	343.1539	343.1545	−1.748483555	C_20_H_22_O_5_	[M+H]^+^	Isoshonanin	Lignans and coumarins	30.00	163.0755; 105.0328; 77.0396; 240.2330; 325.2717
14	9.51	279.1597	279.1596	0.358218023	C_16_H_22_O_4_	[M+H]^+^	Cytosporone C	Others	30.00	149.0238; 121.0285; 57.0700; 65.0384; 93.0337
15	1.20	132.1018	132.1024	−4.541931108	C_6_H_13_NO_2_	[M+H]^+^	6-Deoxyfagomine	Alkaloids	40.00	86.1017; 69.0735; 57.0586; 56.0504; 58.0668
16	3.00	217.0974	217.0977	−1.381866321	C_12_H_12_N_2_O_2_	[M+H]^+^	Lycoperodine-1	Alkaloids	30.00	144.0831; 143.0735; 127.0541; 74.0238; 128.0518
17	0.90	191.0187	191.0192	−2.617537923	C_6_H_8_O_7_	[M-H]^−^	Citric acid	Organic acids	−20.00	111.0088; 87.0085; 85.0278; 111.0020; 129.0181
18	1.00	133.0139	133.0137	1.503604516	C_4_H_6_O_5_	[M-H]^−^	L-Malic acid	Organic acids	−20.00	71.0134; 115.0036; 72.9933; 133.0133; 89.0228
19	7.90	476.2774	476.2777	−0.629884624	C_23_H_42_NO_7_P	[M+H]^+^	LysoPE 18:3	Lipids	30.00	335.2550; 476.2755; 261.2212; 458.2602
20	8.10	452.2773	452.2777	−0.884412386	C_21_H_42_NO_7_P	[M+H]^+^	LysoPE 16:1	Lipids	30.00	311.2614; 452.2752; 280.2671; 219.2082; 237.2180

### 3.3 Network pharmacology analysis

#### 3.3.1 Potential bioactive compounds and targets of *Scrophulariae Radix* in the treatment of anti-neoplastic

In this study, a total of 738 chemical components of *Scrophulariae Radix* were detected using UHPLC-ESI-MS/MS. By searching the TCMSP (https://old.tcmsp-e.com/tcmsp.php) (Ru et al., 2014) and PubChem databases (https://pubchem.ncbi.nlm.nih.gov/) (Wang et al., 2012; Kim et al., 2021), 124 potential bioactive ingredients were filtered out according to Lipinski’s rules of 5 (Lipinski et al., 2001), with the MW not exceeding 500, the XLogP smaller than 5, the H-bond donors less than 5, and the H-bond acceptors less than 10.

#### 3.3.2 Target prediction of *Scrophulariae Radix* and neoplasm-associated target screening

**Figure 10:**
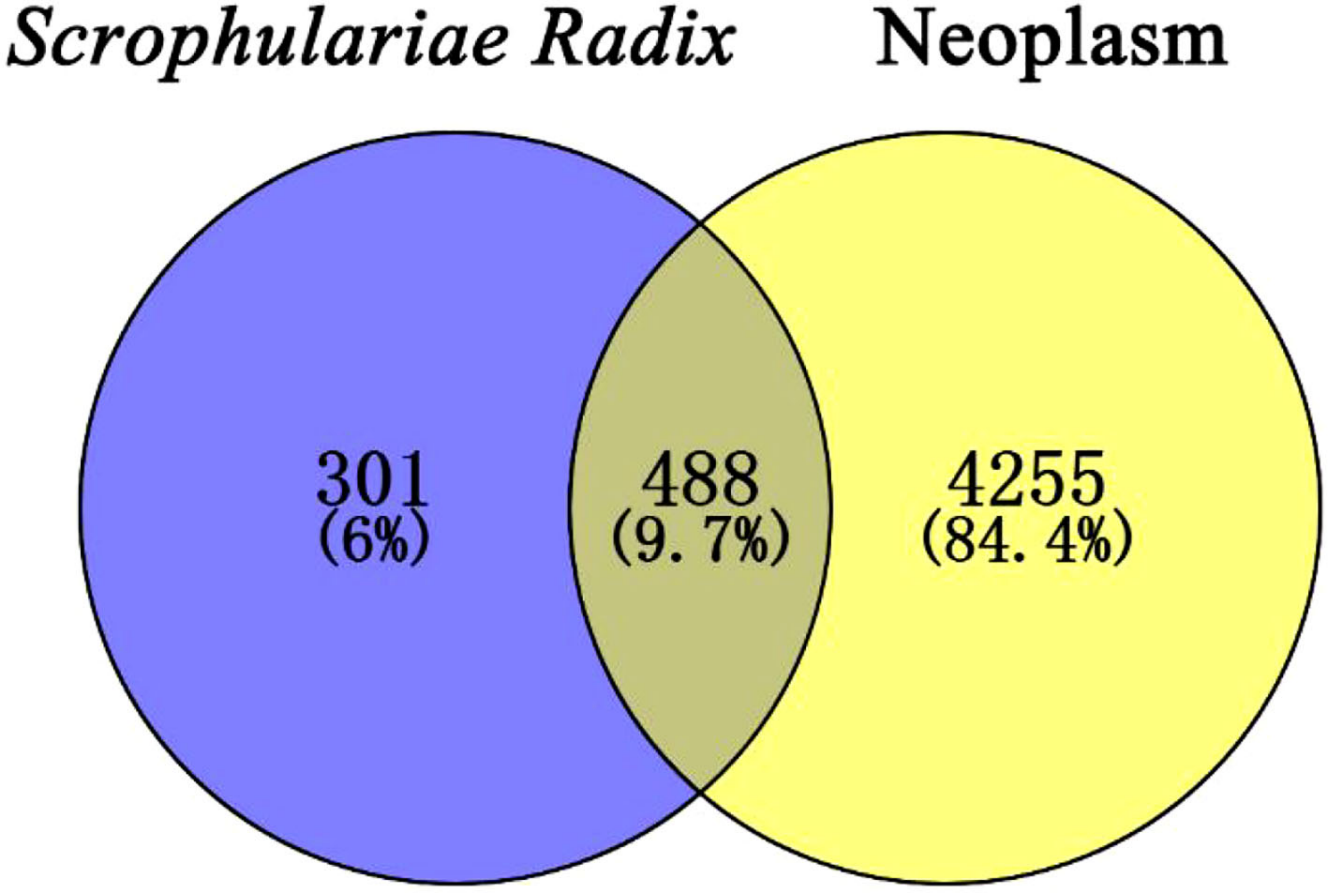
Venn diagram of the 488 common targets between the active compounds’ targets of *Scrophulariae Radix* (SR) and the disease targets of neoplasm.

Target prediction was carried out through the SwissTargetPrediction (https://swisstargetprediction.ch/) (Gfeller et al., 2014; Daina et al., 2019). However, it should be mentioned that six compounds showed no target. The target genes of the remaining 118 compounds were combined. Then, the targets whose probability was greater than that of the median value of 0.1 were selected. After the removal of repetition values, 789 target genes of these compounds were obtained. Using DisGeNET (https://www.disgenet.org/home/) (Piñero et al., 2019), “neoplasm/neoplasm” was input as a keyword, resulting in 10,161 targets, and 4,743 targets with scores greater than the median value of 0.03 were selected. A Venn diagram (**Figure 10**) by Venny 2.1.0 evaluated the coincidence degree between the active components of *Scrophulariae Radix* and neoplasm targets, exhibiting that 488 targets (9.7%) met the conditions.

#### 3.3.3 PPI network of therapeutic targets for *Scrophulariae Radix* against neoplasm

**Figure 11:**
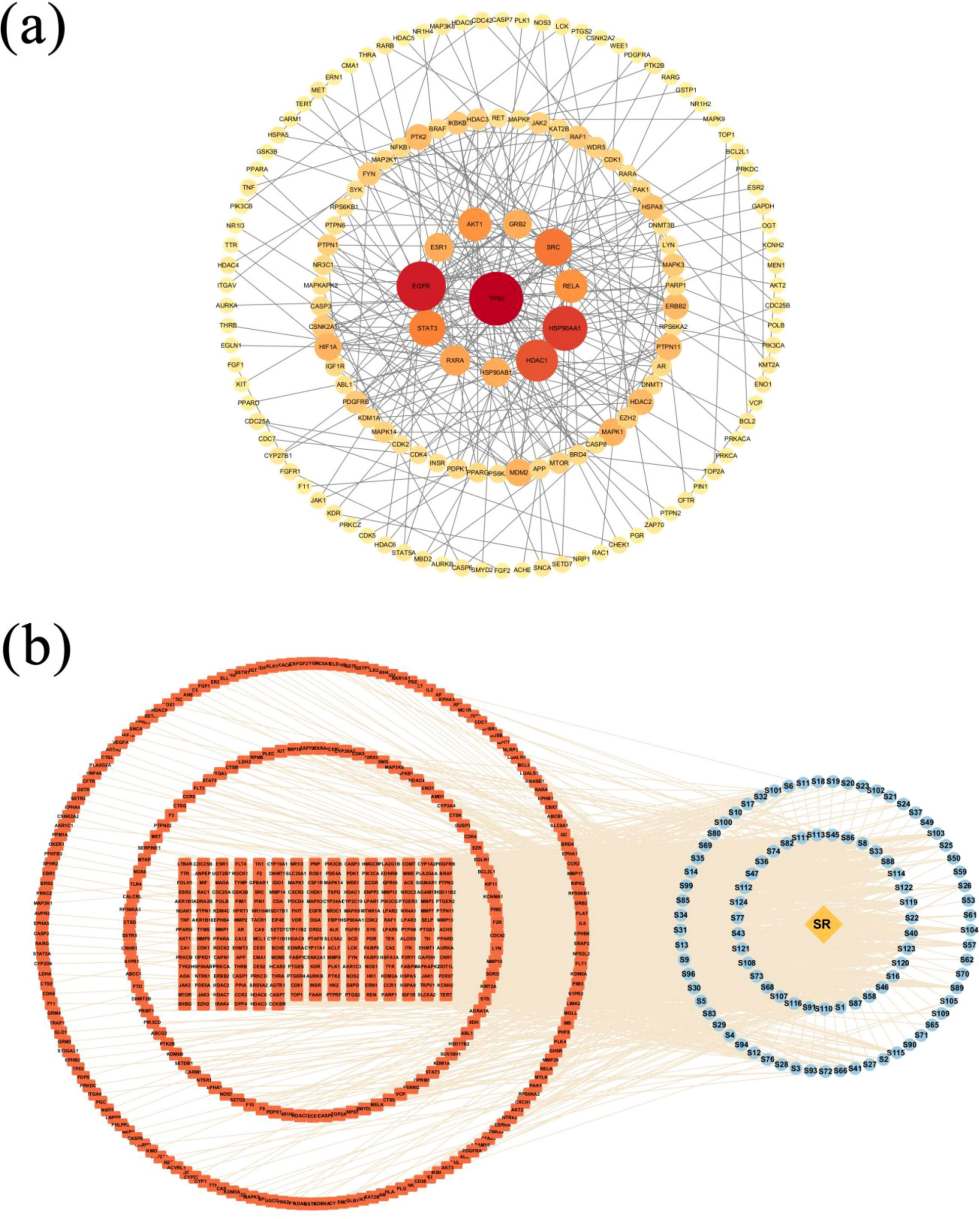
Network construction and analysis results. **(a)** PPI network of the 488 common target genes. Circles represent proteins, the size and the colors (from red to orange to yellow) indicate the degree of binding between proteins, and the lines represent protein–protein interactions. **(b)** The “Drug-Compound-Target” interaction network of *Scrophulariae Radix* for treating neoplasm. Annotation: The yellow diamond represents the drug, the blue ellipses are active compounds, the orange round rectangles are the target genes of the disease, and the lines represent the interactions between compounds and targets.

In total, 488 common targets were input to the String database (https://cn.string-db.org/). Based on a previous study, we set the screening conditions as 0.7 as the minimum required interaction score, degree >40, and betweenness centrality >0.024. Only bioactive interaction sources verified using experiments were selected, and the PPI network was exported as a TSV file to Cytoscape, where the visualization was realized; and the PPI network map of intersecting genes was achieved as shown in **Figure 11a**. The network has 152 nodes and 281 edges, with the nodes suggesting potential targets and edges on behalf of the relation between the two connected targets. Moreover, the size and color were proportional to the degree value of the nodes, with red indicating larger values and yellow indicating smaller values.

#### 3.3.4 “Drug-Compound-Target” network construction and analysis

Using Cytoscape 3.10 (Shannon et al., 2003), the “Drug-Compound-Target” network of *Scrophulariae Radix* for treating neoplasm was built, which contained 585 nodes and 1,898 sides, as shown in **Figure 11b**. The 585 nodes contain 96 compounds that are shown in blue, 488 targets which are displayed in red, and a drug (SR, *Scrophulariae Radix*) which is exhibited in orange. The relationship between drugs, ingredients, and targets was represented by 1,898 edges. To get the core components and targets, nodes were filtered by the CentiScaPe 2.2 plug-in based on degree, betweenness, and closeness centrality. Finally, 44 compounds and 32 targets proved to be critical. The eight compounds with the highest degree are shown in **Table 2**. Moreover, the top 8 targets with the highest degree are shown in **Table 3**.

**Table 2:** Features of the key compounds in the network.

Compound ID	Chemical name	Degree	Betweenness centrality	Closeness centrality
S82	Cytosporone C	74	30,048.38785	6.63E−04
S110	Cystomexicone A	72	22,016.49337	6.61E−04
S86	Mediterraneone	70	23,306.22064	6.59E−04
S73	Bestim	70	46,205.20586	6.59E−04
S114	Hydroxy ricinoleic acid	65	16,962.24961	6.55E−04
S116	Carnosic acid quinone	65	27,574.16306	6.55E−04
S120	Araucarolone	63	14,943.08473	6.53E−04
S108	Negundoin A	62	26,177.67498	6.52E−04

**Table 3:** Topology parameter characteristics of significant genes.

Target name	Gene symbol	Degree	Betweenness centrality	Closeness centrality
CA12	Carbonic anhydrase 12	31	6,109.910978	6.05E−04
CA2	Carbonic anhydrase 2	30	6,726.484516	6.25E−04
CA9	Carbonic anhydrase 9	29	4,811.374483	5.95E−04
CA1	Carbonic anhydrase 1	29	6,624.648575	6.25E−04
FABP3	Fatty acid binding protein 3	19	2,230.032643	5.26E−04
ESR2	Estrogen receptor 2	16	3,240.004779	6.00E−04
MMP1	Matrix metallopeptidase 1	16	4,304.898131	6.35E−04
MMP3	Matrix metallopeptidase 3	15	7,523.994596	6.79E−04

#### 3.3.5 GO pathway enrichment analysis

**Figure 12:**
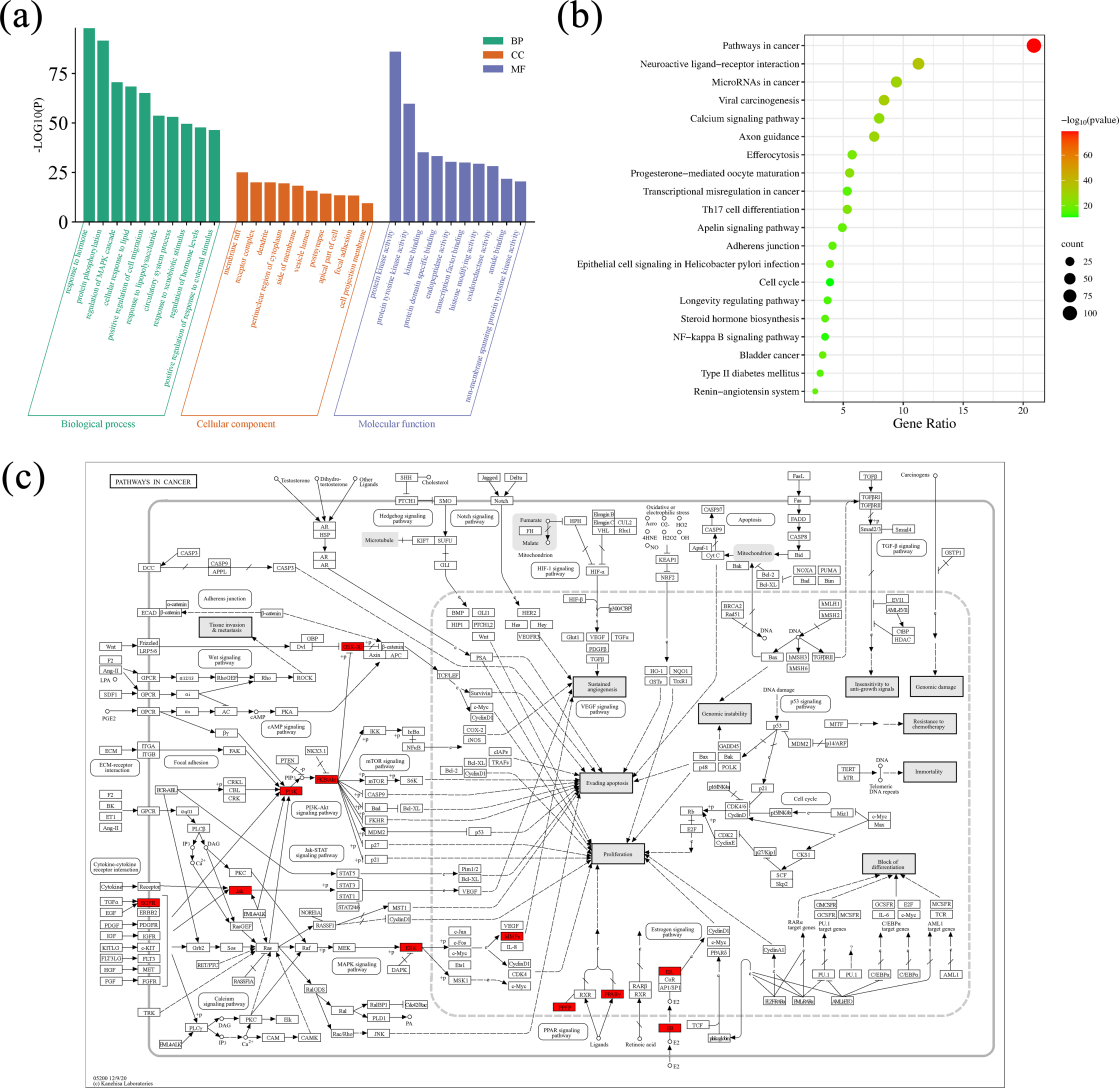
GO and KEGG enrichment analysis results. **(a)** Visualization of GO term enrichment. The green color represents the biological process (BP), the orange color represents the cellular component (CC), and the purple represents the molecular function (MF). **(b)** Visualization of KEGG pathway enrichment. Annotation: Green indicates smaller values and red indicates larger values. **(c)** The action of the core target on the anti-neoplastic signaling pathway. Annotation: Red rectangles represent the key targets.

The Metascape database was adopted for GO enrichment analysis (Ashburner et al., 2000) of 32 potential targets of *Scrophulariae Radix*. A total of 1,555 GO pathways (*p* < 0.01) were enriched, including 1,342 for biological processes (BPs), 104 for cellular components (CCs), and 109 for molecular functions (MFs). **Table 4** and **Figure 12a** show the top 10 ranked enrichment items of BP, CC, and MF, for a total of 30. According to the result, the BP terms were mainly related to cardiac muscle cell development, response to hormones, and protein phosphorylation, and then the CC result revealed that most targets may be located on the membrane raft, receptor complex, and dendrite. Finally, the top 3 MF biological process terms were protein kinase activity, protein tyrosine kinase activity, and kinase binding.

**Table 4:** Terms, descriptions, and symbols of GO enrichment pathways.

Category	Pathway ID	Pathway name	−Log10(*P*)	Count
Biological process	GO:0055013	Cardiac muscle cell development	97.85	134
Biological process	GO:0009725	Response to hormone	91.57	109
Biological process	GO:0006468	Protein phosphorylation	70.50	104
Biological process	GO:0043408	Regulation of MAPK cascade	68.40	94
Biological process	GO:0071396	Cellular response to lipid	65.13	94
Biological process	GO:0030335	Positive regulation of cell migration	53.66	68
Biological process	GO:0032496	Response to lipopolysaccharide	52.98	79
Biological process	GO:0003013	Circulatory system process	49.49	71
Biological process	GO:0009410	Response to xenobiotic stimulus	47.72	76
Biological process	GO:0010817	Regulation of hormone levels	46.43	76
Cellular component	GO:0045121	Membrane raft	25.00	43
Cellular component	GO:0043235	Receptor complex	19.91	50
Cellular component	GO:0030425	Dendrite	19.91	53
Cellular component	GO:0048471	Perinuclear region of the cytoplasm	19.48	57
Cellular component	GO:0098552	Side of the membrane	18.24	55
Cellular component	GO:0031983	Vesicle lumen	15.73	35
Cellular component	GO:0098794	Postsynapse	14.28	46
Cellular component	GO:0045177	Apical part of the cell	13.37	38
Cellular component	GO:0005925	Focal adhesion	13.31	36
Cellular component	GO:0031253	Cell projection membrane	9.36	28
Molecular function	GO:0004672	Protein kinase activity	85.99	110
Molecular function	GO:0004713	Protein tyrosine kinase activity	59.68	54
Molecular function	GO:0019900	Kinase binding	35.13	75
Molecular function	GO:0019904	Protein domain-specific binding	33.19	67
Molecular function	GO:0004175	Endopeptidase activity	30.34	51
Molecular function	GO:0008134	Transcription factor binding	29.95	61
Molecular function	GO:0140993	Histone modifying activity	29.30	39
Molecular function	GO:0016705	Oxidoreductase activity, acting on paired donors, with incorporation or reduction of molecular oxygen	28.13	36
Molecular function	GO:0033218	Amide binding	21.69	43
Molecular function	GO:0004715	Non-membrane spanning protein tyrosine kinase activity	20.35	18

#### 3.3.6 KEGG pathway enrichment analysis

A total of 32 core targets were identified through KEGG pathway enrichment analysis (Kanehisa and Goto, 2000; Kanehisa et al., 2017). Then, parameter conditions of terms with a *p*-value <0.01, a minimum count of 3, and an enrichment factor >1.5 were collected and grouped into clusters based on their membership similarities. The top 20 pathways ranked in terms of *p*-value are exhibited in **Table 5**, and the top 20 pathways are visualized in **Figure 12b**. The size of the circle suggested the number of correlated genes in the pathway, and the color indicated the *p*-value. Therefore, the network’s top 5 remarkable KEGG pathways include mainly pathways in neoplasm (hsa05200) (**Figure 12c**), neuroactive ligand–receptor interaction pathway (hsa04080), viral carcinogenesis pathway (hsa05203), microRNAs in the neoplasm pathway (hsa05206), and axon guidance pathway (hsa04360), which indicated that *Scrophulariae Radix* might exert its antitumor effect potentialities by these multiple pathways.

**Table 5:** Top 20 KEGG pathways ranked by *p*-value.

Category	Pathway ID	Pathway name	*p*-value	Count
KEGG pathway	hsa05200	Pathways in cancer	2.19E−79	102
KEGG pathway	hsa04080	Neuroactive ligand–receptor interaction	1.66E−36	55
KEGG pathway	hsa05203	Viral carcinogenesis	1.05E−32	41
KEGG pathway	hsa05206	MicroRNAs in cancer	1.86E−30	46
KEGG pathway	hsa04360	Axon guidance	8.32E−30	37
KEGG pathway	hsa04020	Calcium signaling pathway	1.35E−26	39
KEGG pathway	hsa04914	Progesterone-mediated oocyte maturation	2.24E−25	27
KEGG pathway	hsa04659	Th17 cell differentiation	2.57E−23	26
KEGG pathway	hsa04148	Efferocytosis	2.75E−21	28
KEGG pathway	hsa05219	Bladder cancer	1.15E−18	16
KEGG pathway	hsa05120	Epithelial cell signaling in *Helicobacter pylori* infection	1.82E−18	19
KEGG pathway	hsa04614	Renin–angiotensin system	4.17E−18	13
KEGG pathway	hsa04371	Apelin signaling pathway	5.13E−18	24
KEGG pathway	hsa04520	Adherens junction	3.39E−17	20
KEGG pathway	hsa00140	Steroid hormone biosynthesis	9.55E−17	17
KEGG pathway	hsa05202	Transcriptional misregulation in cancer	1.17E−16	26
KEGG pathway	hsa04930	Type II diabetes mellitus	3.39E−16	15
KEGG pathway	hsa04211	Longevity regulating pathway	4.17E−15	18
KEGG pathway	hsa04064	NF-kappa B signaling pathway	1.12E−12	17
KEGG pathway	hsa04110	Cell cycle	1.10E−11	19

#### 3.3.7 Molecular docking verification

**Figure 13:**
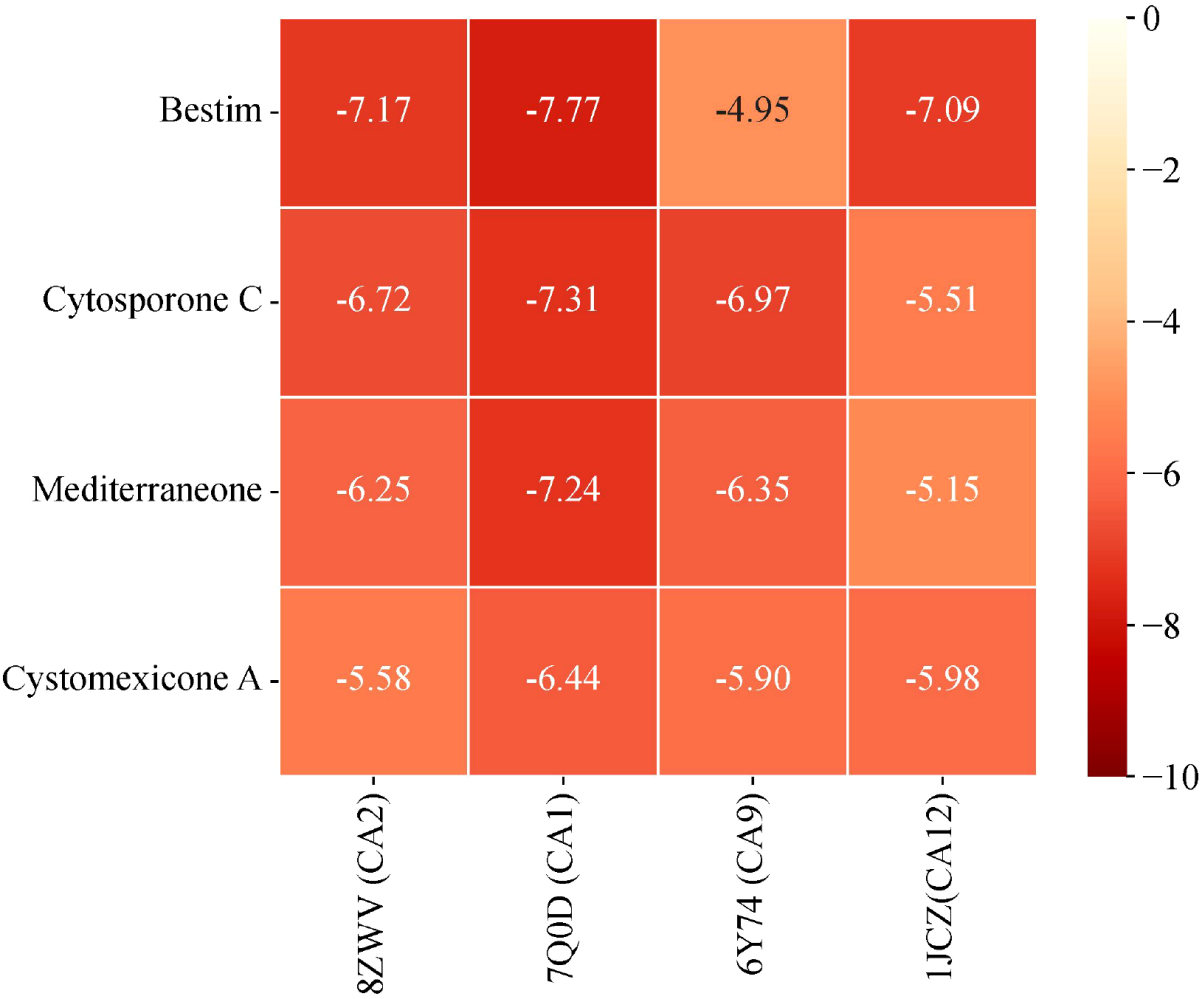
Heatmap of the binding energy matrix for molecular docking of core compounds with gene targets.

**Figure 14:**
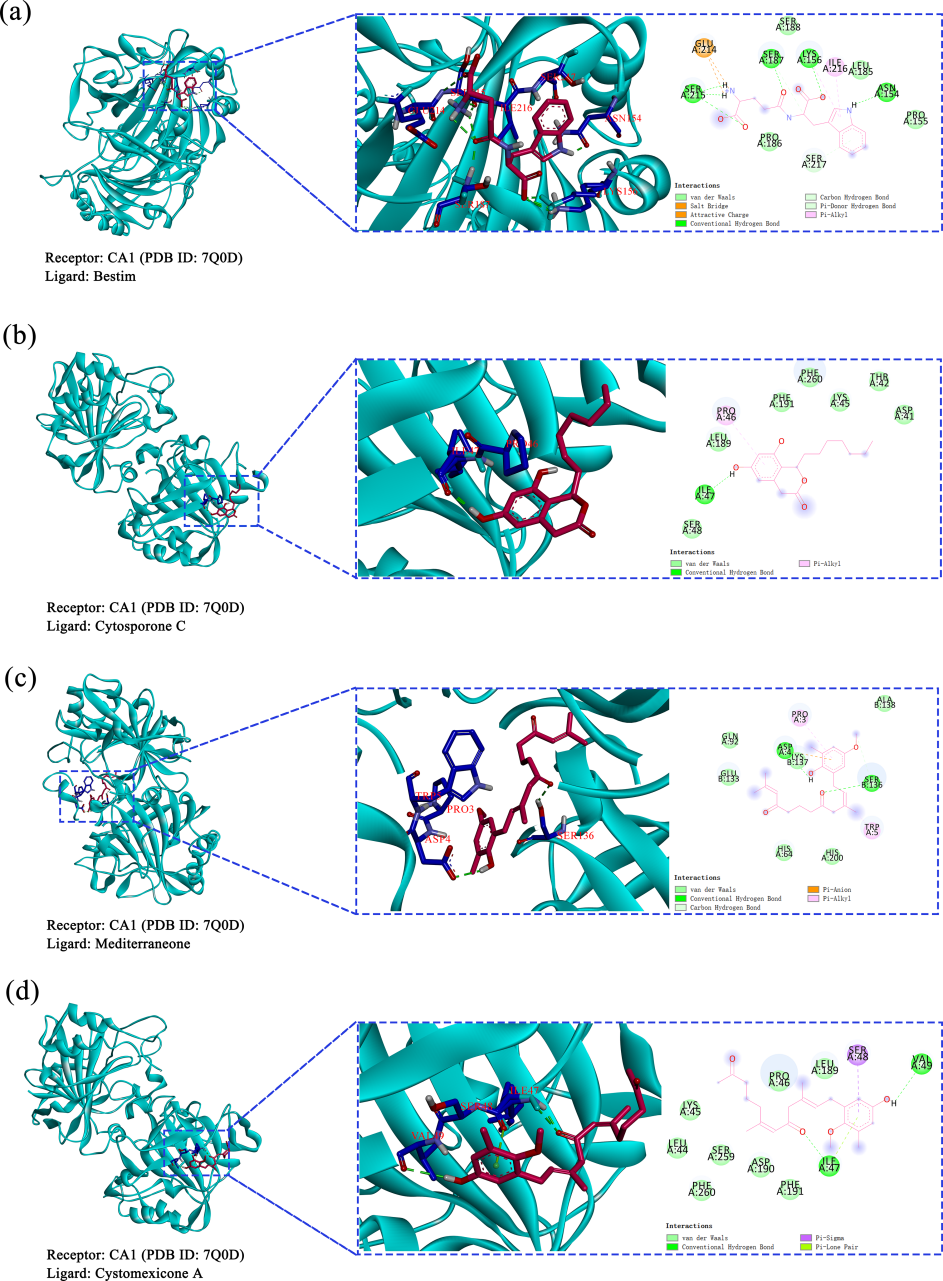
Molecular docking results of core compounds and corresponding proteins of gene targets: **(a)** 7Q0D-bestim, **(b)** 7Q0D-cytosporone C, **(c)** 7Q0D-mediterraneone, and **(d)** 7Q0D-cystomexicone A.

Based on the predictions from network pharmacology, cytosporone C, cystomexicone A, mediterraneone, and bestim were selected as the four core compounds for this study due to their significant association with key targets, as indicated by their notable centrality in CentiScaPe 2.2. Moreover, they were considered to be key targets of potential targets for cancer treatment (CA12, CA2, CA9, and CA1), as verified by molecular docking analysis and binding energy calculation, visualized through a heatmap generated on the microscopic map matrix heat map letter platform (https://www.bioinformatics.com.cn), as shown in **Figure 13**. Therefore, to verify the potential role of *Scrophulariae Radix* in neoplasm treatment, the binding energy calculated by AutoDock Vina was applied to evaluate the bonding bioactivity between the docking molecules, with a smaller Vina score implying a stronger bonding bioactivity, a higher affinity, and a more stable structure between the ligand and receptor. Compared to a control medicine, these significant constituents displayed a stronger affinity for the crucial targets (< -5.0 kcal/mol, **Figure 13**), which proved favorable docking and high affinity. Hence, we finally chose the top 4 Vina scores for each significant constituent: (A) 7Q0D-bestim, (B) 7Q0D-cytosporone C, (C) 7Q0D-mediterraneone, and (D) 7Q0D-cystomexicone A, respectively, and then their selected targets were visualized by Discovery Studio 2019 in **Figure 14**. The analysis results exhibited that the binding energies of the four groups were −7.77 kcal/mol, −7.31 kcal/mol, −7.24 kcal/mol, and −6.44 kcal/mol, respectively, which were all lower than −5 kcal/mol, indicating that the docking point and structure of small-molecule ligands and protein receptors were stable.

#### 3.3.8 Molecular dynamics simulations and binding free energy calculations

**Figure 15:**
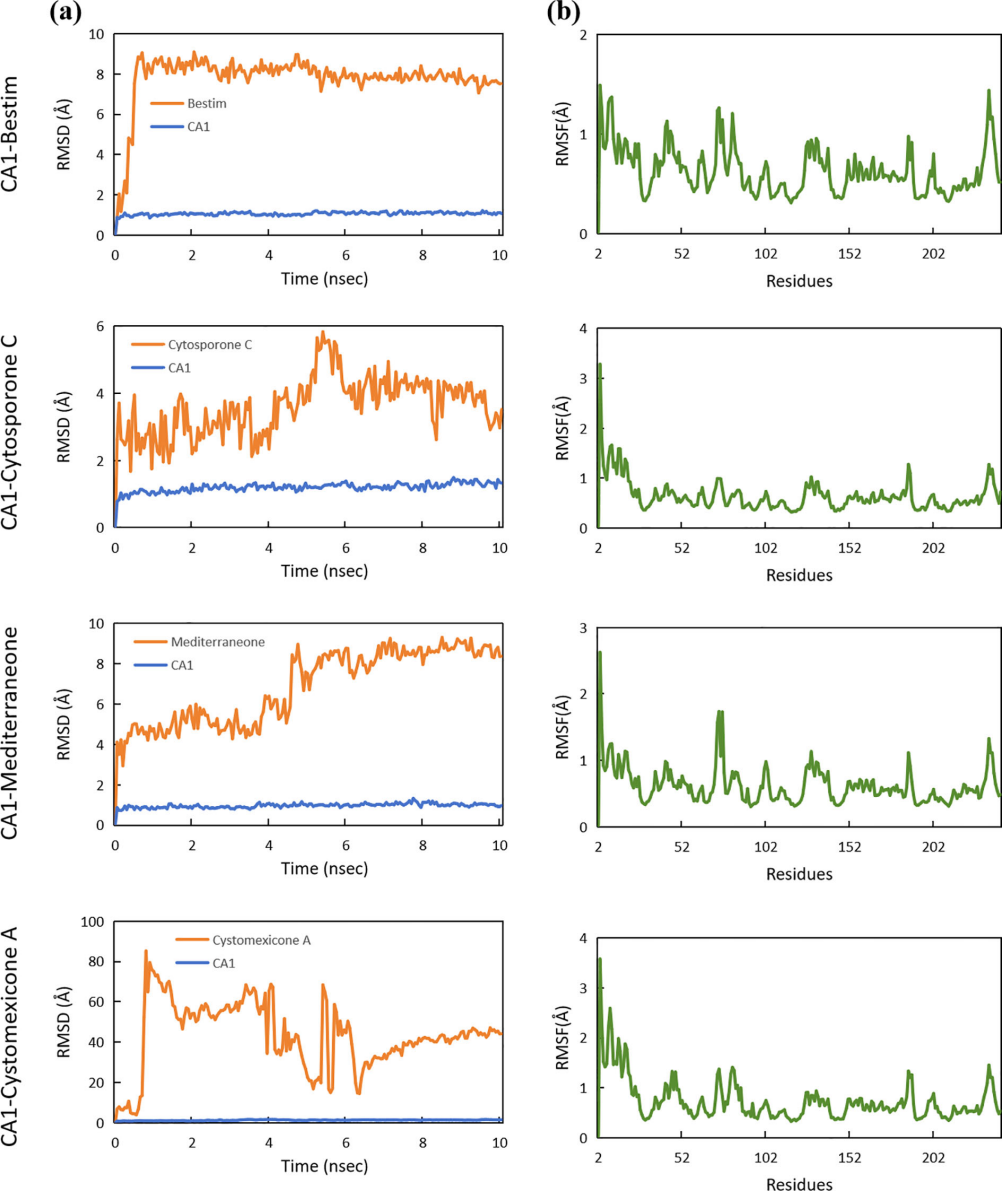
**(a)** RMSD analysis and **(b)** RMSF analysis of molecular dynamics for the CA1 protein with bestim, cytosporone C, mediterraneone, and cystomexicone A ligands.

To further investigate the stability and interactions of the complexes, molecular dynamics simulations were conducted. The RMSD was utilized to measure the average change in displacement of a selection of atoms for a particular frame with respect to a reference frame. The stability of the systems was measured by comparing the RMSD of the ligand and protein, where a lower RMSD signifies enhanced stability. As depicted in **Figure 15a**, the RMSD values for the four proteins were generally approximately 1.00 Å, which validated the stability of the CA1 protein. Notably, the RMSD values of the CA1 protein in the CA1-mediterraneone complex was 0.96 ± 0.12 Å, with minor fluctuations, confirming the binding stability of mediterraneone with CA1. Furthermore, the stability of the ligands within the binding site was examined. The compound cytosporone C exhibited stable interactions with CA1, with an RMSD of 3.60 ± 0.88 Å. However, cystomexicone A displayed a large deviation during molecular dynamics simulations, reaching an RMSD of 43.08 ± 17.01 Å. In terms of flexibility, RMSF analysis was conducted. With the exception of the expected fluctuations in the C-terminal, the RMSF values of the CA1 residues remained below 2.00 Å throughout the simulation, as depicted in **Figure 15b**. It indicated that the CA1 protein maintained structural stability when bound to the four ligands.

**Figure 16:**
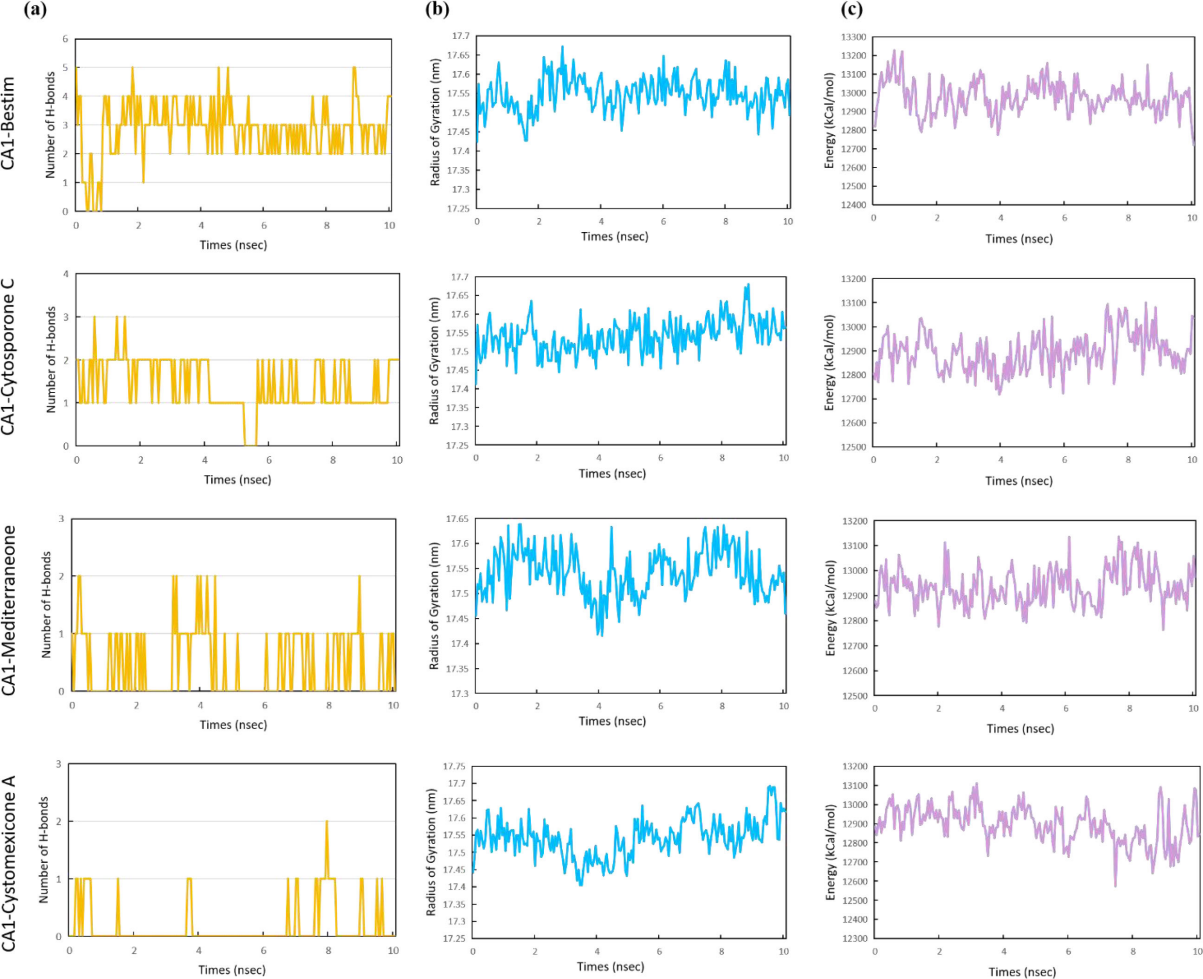
The number of H-bonds **(a)**, radius of gyration **(b)**, and energy **(c)** for the CA1 protein with bestim, cytosporone C, mediterraneone, and cystomexicone A ligands over 10 ns molecular dynamics simulations.

In addition, the number of H-bonds, radius of gyration (Rg), and energy were evaluated for the four complexes to assess their stability and binding characteristics. The number of H-bond plots revealed that the compound bestim formed more hydrogen bonds with CA1 compared to other compounds, maintaining two or four hydrogen bonds during 1–10 ns (**Figure 16a**). The radius of gyration (Rg) is an indicator of protein compactness, with lower values indicating a more compact and rigid conformation. As illustrated in **Figure 16b**, the four complexes exhibited a similar degree of compactness, with average Rg values approximately 17.54 nm. Likewise, the energy plots showed that the CA1 protein exhibited an average energy of approximately 12,900.00 kcal/mol across four complexes (**Figure 16c**). Among all complexes, the CA1-cytosporone C complex had the lowest average Rg of 17.541 nm and energy of 12,894.09 kcal/mol. In summary, the MD simulation results indicated that the CA1-cytosporone C complex displays favorable structural stability and can be further explored.

## 4 Discussion

The current study combined the response surface method, UPLC-ESI-MS/MS, network pharmacology, molecular docking, and MD simulation to characterize the active constituents in *Scrophulariae Radix*, so as to further explore and uncover their potential mechanisms against neoplasm.

According to the National Cancer Registry in the World Health Organization, neoplasm is the most frequently diagnosed cancer (Khoury et al., 2022). As a leading cause of mortality worldwide, neoplasm remains difficult to treat due to its considerable confusion and extremely rapid spread, making disease control difficult (Ruirui et al., 2021; Miao et al., 2023). Unfortunately, the world is currently faced with a series of challenges for neoplasm treatment, such as drug resistance caused by chemotherapy, post-surgical resection recurrence, and infection (Ma et al., 2019; Miao et al., 2023). As a result, there is a pressing demand for the development of efficacious treatment options to combat neoplasm. Thus, scientific research endeavors aimed at the exploration of new therapeutic target drugs and innovative approaches to enhance existing treatments have been at the forefront (Huang et al., 2020).

Currently, the utilization of TCM for neoplasm treatment is widely acknowledged and has garnered significant attention from researchers (Pangestuti and Kim, 2011). TCM comprises numerous bioactive constituents that have shown potential in neoplasm treatment (Ma et al., 2019; Li et al., 2022b; Zhai et al., 2022; Zhang et al., 2022). For example, previous research has investigated the anti-neoplasm effects of *Scrophulariae Radix* (Kim et al., 2017). With continued research, the extractions of *Scrophulariae Radix* have been adopted for the treatment of neoplasm and exhibited remarkable and potent antitumor effects (Qi et al., 2016; Baradaran et al., 2017). These pharmacodynamic bioactivities might be related to the iridoid glycosides and phenylpropanoid glycosides derived from *Scrophulariae Radix*. Currently, the research focused on characterizing the bioactive constituents of *Scrophulariae Radix* with low content but significant potential, aiming to establish it as a novel and effective treatment option against cancer (Lu et al., 2019). Nevertheless, due to the highly heterogeneous and diverse structures of iridoid/phenylpropanoid glycosides, substantial iridoid/phenylpropanoid glycosides that might show as low-abundance/trace species need to be explored. Thus, the challenges for the detection of low-abundance iridoid/phenylpropanoid are mainly derived from the ion suppression of co-eluted species. Particularly, the interfering signals arising from the matrix, contaminants, and isomers might compromise the MS/MS spectra of iridoid/phenylpropanoid glycosides, thus hindering the identification of iridoid/phenylpropanoid glycosides. To address these challenges, we herein developed a response surface analysis procedure prior to LC-MS analysis of iridoid/phenylpropanoid glycosides.

Initially, to explain the TCM interactions that occur when extracts from these TCMs are achieved together, the response surface analysis method is a modern optimization technique that presents several advantages over traditional methods, including the ability to simultaneously change multiple factors and acquire the effects of interactive factors (Bohlooli et al., 2017). Several successful examples of response surface analysis applications in TCM could be found in previous reports (Yuan et al., 2018). Thus, the optimum extraction condition of *Scrophulariae Radix* was significantly improved, and the material–liquid ratio was reduced from 100 g/mL to 32 g/mL. Meanwhile, combining response surface analysis with UPLC-ESI-MS is a common technique for drug metabolism and has been widely employed to measure the bioactive constituents of TCM (Guo et al., 2024). By using this improved approach, a total of 738 compounds, including 161 terpenoids, 144 phenolic acids, 51 alkaloids, 24 flavonoids, and 34 saccharides, were characterized and elucidated from *Scrophulariae Radix*. This number was almost five of that of a previous publication (You-Hua et al., 2014). In particular, a significant improvement in the characterization of terpenoids, phenolic acids, and saccharides was obtained, especially species in organic acids and alkaloids. This integration method was demonstrated to be vital for the accurate analysis of natural products, particularly those low-abundance species. Nevertheless, which constituents are most effective, the potential target, and the action mechanisms are not fully understood, which limits the application of *Scrophulariae Radix* (Lu et al., 2019).

In recent years, network pharmacology and molecular docking have usually been used to reveal active constituents and the molecular action mechanisms of disease treatment and elucidate the interactions of compound targets (Li and Zhang, 2014; Sun et al., 2023). In the current study, the UPLC-MS/MS analysis identified 738 compounds, and 124 of them followed the standards of Lipinski’s rules of 5. A total of 124 constituents remained more likely to be taken orally. Then, these constituents corresponded to 488 anti-neoplasm targets. Different constituents might synergistically participate in anti-neoplastic through regulating the corresponding targets and signaling pathways. Four of the 124 constituents were selected for molecular docking based on their anti-neoplasm targets exceeding the mean value.

Among them, 488 key anti-neoplasm targets and 486 were identified via the String database. Only active interaction sources verified by experiments were selected, and 0.7 was set as the minimum required interaction score. Finally, the network established included 182 nodes when the PPI network data were imported into Cytoscape 3.1. Of the 182 network nodes, 30 nodes were independent. Thus, those 30 independent nodes were removed. Herein, 152 targets and 281 edges were achieved, with the nodes suggesting potential targets and edges on behalf of the relation between the two connected targets.

Based on the above results, Cytoscape was applied to construct the “Drug-Compound-Target” network of *Scrophulariae Radix* for treating neoplasms, and 44 components and 32 targets were ultimately thought of as critical. The four compounds with the highest degree were cytosporone C, cystomexicone A, mediterraneone, and bestim. The top 4 targets with the highest degree were carbonic anhydrase 12 (CA12), carbonic anhydrase 2 (CA2), carbonic anhydrase 9 (CA9), and carbonic anhydrase 1 (CA1).

The enrichment analysis included biological processes (BPs), cellular components (CCs), molecular functions (MFs), and KEGG analysis. The top 10 CCs displayed that the 32 core targets were prominently enriched in different cellular components, including the membrane raft, receptor complex, and dendrite. A variety of CCs revealed that these constituents might trigger multi-BP at different cellular sites. The top 3 MFs were related to the binding and activity of protein kinase activity, protein tyrosine kinase activity, and kinase binding. Hence, we speculated that *Scrophulariae Radix* may achieve its antitumor effect through the above pathways. Meanwhile, the anti-neoplasm mechanism of the screened constituents might regulate the transcription and modifications of neoplasm-relevant proteins.

KEGG pathway enrichment results exhibited that the five core channels associated with the anti-neoplasm activity of *Scrophulariae Radix* were the pathways in cancer (hsa05200), neuroactive ligand–receptor interaction (hsa04080), viral carcinogenesis (hsa05203), microRNAs in cancer (hsa05206), and axon guidance (hsa04360). The top KEGG signaling pathway enriched by the key targets of the four constituents is closely related to neoplasm. By targeting the key targets, the four constituents of *Scrophulariae Radix* are promising in combating neoplasm.

Though network pharmacology offers an assessment of constituent suitability in the protein bioactive site, it merely offers information at the protein active site (Wang et al., 2022). Thus, to further estimate the compound–protein target system, the application of binding conformation information has already become more widespread and requires the application of molecular docking and molecular dynamics (MD) simulations, as well as their related binding energy measurements (Zhou et al., 2022). Molecular docking showed that the binding energies of the four ligand–protein groups were −7.77 kcal/mol, −7.31 kcal/mol, −7.24 kcal/mol, and −6.44 kcal/mol, respectively. Then, MD simulations promote in-depth exploration of the dynamic properties of the docked complexes and fluctuations in the energy action. RMSD was used to measure the average displacement change of selected atoms relative to a reference frame. Lower RMSD values indicated greater system stability. The RMSD values of the four proteins were approximately 1.00 Å, confirming the stability of the CA1 protein. The CA1-mediterraneone complex had an RMSD of 0.96 ± 0.12 Å, showing stable binding. Cytosporone C had an RMSD of 3.60 ± 0.88 Å, indicating stable interactions with CA1, while cystomexicone A had a large RMSD deviation of 43.08 ± 17.01 Å. The RMSF analysis showed that CA1 residues remained stable, with values below 2.00 Å, except for the expected C-terminal fluctuations.

In a word, our investigation has established a foundational framework for elucidating the multiconstituent, multitarget, and multisignaling pathway effects of *Scrophulariae Radix* as a potential therapeutic agent for neoplasm treatment. By integrating network pharmacology and bioinformatics approaches, we have successfully identified key interactions and molecular pathways that underpin the antineoplastic activity, thereby facilitating the characterization of prospective medicine targets to combat this disease.

## 5 Conclusions

This study combined response surface analysis, UPLC-ESI-MS/MS, network pharmacology, molecular docking, and molecular dynamics simulations to characterize active constituents in *Scrophulariae Radix* and explore its anti-neoplastic mechanisms. The experimental extraction efficiency of six reference standards was 1.332%, aligning well with the predicted value of 1.346% from response surface analysis, indicating a well-fitted model. A total of 738 compounds were identified from *Scrophulariae Radix* for the first time. Cytosporone C, cystomexicone A, mediterraneone, and bestim were the top 4 active compounds with the highest connectivity degrees, corresponding to key targets such as CA12, CA2, CA9, and CA1. Molecular docking showed binding energies of −7.77 kcal/mol, −7.31 kcal/mol, −7.24 kcal/mol, and −6.44 kcal/mol for the four ligand–protein complexes. Molecular dynamics simulations confirmed the stability of the docking results, highlighting *Scrophulariae Radix* as a promising anti-neoplastic drug candidate, offering additional scientific support for the clinical application of *Scrophulariae Radix* in neoplasm treatment.

## Data Availability

The original contributions presented in the study are included in the article/**Supplementary Material**. Further inquiries can be directed to the corresponding authors.
